# Environmental Control of Ferroelectricity in Hafnia Films

**DOI:** 10.1002/adma.202503852

**Published:** 2025-08-07

**Authors:** Waseem Ahmad Wani, Nicolas K. Lam, Kristina M. Holsgrove, Gerald Bejger, Tinsae Alem, Kory Burns, Stephen J. McDonnell, Christina M. Rost, Amit Kumar, Jon F. Ihlefeld, Brian J. Rodriguez

**Affiliations:** ^1^ School of Physics and Conway Institute University College Dublin Belfield Dublin 4 Ireland; ^2^ Department of Materials Science and Engineering University of Virginia Charlottesville VA 22904 USA; ^3^ Centre for Quantum Materials and Technologies, School of Mathematics and Physics Queen's University Belfast Belfast BT7 1NN UK; ^4^ Department of Materials Science and Engineering Virginia Polytechnic Institute and State University Blacksburg VA 24061 USA; ^5^ Charles L. Brown Department of Electrical and Computer Engineering University of Virginia Charlottesville VA 22904 USA

**Keywords:** domain retention, electrochemical states, hafnia, oxygen vacancies, PFM

## Abstract

Ferroelectricity in hafnia films has triggered significant research interest over the past decade due to its immense promise for next‐generation memory devices. However, the origin of ferroic behavior at the nanoscale and the means to control it remain an open question, with the consensus being that it deviates from conventional ferroelectrics. In this work, a novel approach is presented to tune ferroelectric properties of hafnia through environmental control using piezoresponse force microscopy (PFM). A reversible transition from non‐ferroelectric to ferroelectric behavior by modulating the surrounding atmosphere is demonstrated. Notably, the domain relaxation dynamics exhibit striking sensitivity to environmental factors, including ambient conditions, specific gas compositions (N_2_, CO_2_, O_2_), and humidity levels. The critical role of surface water removal, gas molecule adsorption, and their interactions with near‐surface oxygen vacancies is identified and the injected charge in determining ferroelectricity in uncapped hafnia films. These insights reveal a significant strategy for stabilizing ferroic responses by carefully regulating the chemical environment, offering new possibilities for precise control in hafnia‐based films.

## Graphical abstract

A reversible transition from nonferroelectric to ferroelectric behavior is achieved in hafnia films by modulating the surrounding atmosphere. The domain relaxation dynamics exhibit high sensitivity to environmental factors, including gas composition (ambient, N_2_, CO_2_, O_2_) and relative humidity, highlighting the crucial role of the measurement environment in determining the ferroelectric properties of hafnia films.

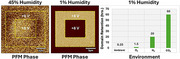

## 1 Introduction

Since ferroelectrics were first proposed as promising candidates for non‐volatile random‐access memory (FeRAM) applications, extensive efforts have focused on their miniaturization for use in ultra‐dense oxide electronics.^[^ ^1^, ^2^, ^3^ ^]^ Despite significant progress in scaling down conventional ferroelectric materials for device applications, commercialization is hindered by size‐related effects and incompatible fabrication methods. However, the discovery of ferroelectricity in hafnia (HfO_2_) has grabbed keen attention in the ferroelectric community due to several promising reasons.^[^ ^1^, ^4^ ^]^ First, hafnium oxide and the various dopants or alloying compounds are compatible with the existing complementary metal oxide semiconductor (CMOS) technology, and amorphous hafnium oxide has already replaced silicon dioxide (SiO_2_) in advanced CMOS technology, enabling the transition to smaller, more efficient devices. Second, hafnia‐based films maintain strong ferroelectric properties even at very small scales (≈1 nm‐thick films).^[^ ^4^, ^5^, ^6^, ^7^ ^]^ As well, hafnia‐based films are heralded for their low power consumption and fast switching speeds.^[^ ^1^, ^8^, ^9^ ^]^ These unique features have invigorated interest in hafnia‐based ferroelectric materials, bringing them back to the forefront of memory technologies.^[^ ^2^, ^10^, ^11^ ^]^

Driven by these unusual features, the polymorphism and ferroelectric response of hafnia films have been linked to various factors, including preparation techniques, chemical make‐up, annealing conditions, surface treatments, field‐induced phase transitions, and changes in oxygen stoichiometry.^[^ ^12^, ^13^, ^14^ ^]^ Several studies suggest that oxygen vacancies are crucial in stabilizing the metastable ferroelectric orthorhombic phase (space group *Pca*2_1_) and enhancing ferroelectricity.^[^ ^13^, ^14^, ^15^, ^16^, ^17^ ^]^ These vacancies stabilize the orthorhombic phase and reduce the energy barrier between the non‐polar and polar phases. Moreover, the redistribution of oxygen vacancies has been proposed as a mechanism for enhancing ferroelectricity through the “wake‐up” effect.^[^ ^16^, ^18^, ^19^ ^]^ These factors contribute to the complexities of stabilizing the ferroelectric orthorhombic phase and in determining the local ferroelectric response; hence, these challenges have slowed progress in achieving phase uniformity across large scales.

Significant progress has already been made toward the development of high‐speed, low‐power, non‐volatile memory and understanding the fundamental physics behind hafnia‐based materials.^[^ ^2^, ^20^, ^21^, ^22^ ^]^ Nevertheless, the ferroelectric behavior of these materials is still not fully understood due to complex structural and chemical factors that control the ferroelectricity in these materials. For example, one key characteristic of hafnia‐based ferroelectrics is the wake‐up effect, wherein ferroelectricity is enhanced through repeated electric field cycling, highlighting their unconventional behavior compared to typical ferroelectrics.^[^ ^16^, ^19^, ^23^ ^]^ In further contrast to traditional ferroelectrics, where dipole moments arise from shifts in cations, in hafnia‐based materials, the oxygen anions shift, making the orthorhombic polar phase highly sensitive to oxygen vacancies and defects. Also, in conventional ferroelectrics, domain switching is typically easier in ambient conditions than under ultra‐high vacuum (UHV). In ambient conditions, the adsorbed water interacts with the ferroelectric surface and generates OH^‒^ molecules and H⁺ ions.^[^ ^24^, ^25^, ^26^, ^27^ ^]^ These species play a crucial role in screening the depolarization field and stabilizing ferroelectric polarization in the absence of electrodes capable of supplying free charge. In contrast, hafnia‐based films exhibit improved stability in UHV conditions but reduced stability in atmospheric environments. Recently, Wei et al. revealed a strong dependence of polarization stability in Hf_0.5_Zr_0.5_O_2_ (HZO) films on atmospheric humidity (ambient with different relative humidity (RH)), reporting a marked decrease in upward polarization stability with increasing humidity.^[^ ^28^ ^]^ This behavior was attributed to the adsorption of water molecules on the film surface, which induces a built‐in electric field directed towards the bottom electrode, thereby influencing the stability and retention of ferroelectric domains.^[^ ^28^ ^]^ Moreover, Zhu et al. conducted time‐dependent polarization relaxation measurements on bare HZO films of varying thicknesses, ranging from 11 to 55 nm.^[^ ^29^ ^]^ They observed that the polarization relaxation process accelerates as the film thickness decreases. This enhanced relaxation behavior with reduced film thickness was attributed to the influence of interface‐bound charges. Kelley et al. demonstrated that the ferroelectric properties of hafnia are highly sensitive to the external environment (ambient vs UHV).^[^ ^30^ ^]^ Their findings confirmed that the electrochemical state of the surface is strongly influenced by the atmospheric partial pressure (i.e., oxygen), which in turn dictates the phase transition from antiferroelectric to ferroelectric in these materials. These observations were interpreted within the framework of an anti‐ferroionic model, which links antiferroelectric bulk behavior with surface electrochemical phenomena. Interestingly, another study argued that atmospheric ions can penetrate the capping electrode layer, directly influencing the ferroelectric properties of these materials.^[^ ^31^ ^]^ A more recent study showed that the piezoresponse in HZO films is not constant but can be modulated when subjected to different AC field cycling.^[^ ^21^ ^]^ Therefore, the interplay of surface electrochemistry, oxygen vacancies, and measurement parameters could be the reason why the piezoelectric response in HZO thin films (capped or bare films) has been the subject of ongoing debate within the scientific community. Some studies report excellent PFM response under ambient conditions, while others have observed no significant PFM response in such conditions (Table S1, Supporting Information). In fact, switching response and stability are better under UHV and low‐humidity environments. However, in these conditions where the moisture and gas environment are controlled, the underlying cause of this improvement compared to conventional ferroelectrics (which typically show better response in ambient conditions) remains unclear. Though a few studies have investigated the role of moisture on the local ferroelectric response of HZO films, there has been no systematic investigation on how different surrounding gases, under varying humidity levels, affect domain stability. Moreover, understanding the intrinsic surface behavior, especially in the absence of a top electrode (which offers protection to the ferroelectric layer), is fundamentally important for gaining deeper insights into stability and device reliability. This highlights the need for a detailed investigation on the impact of environmental control and choice of measuring parameters on retention properties of HZO, as understanding and addressing these interconnected factors is crucial to resolving the discrepancies in the ferroelectric response reported for uncapped HZO thin films.

To address these inconsistencies and gain deeper insight into the underlying mechanisms, we systematically examined how the measurement environment (including gas composition, humidity, and their combination) affects the ferroelectric behavior of HZO thin films. Initially, we investigated the impact of humidity, followed by a controlled exploration of domain relaxation in different gaseous environments, including ambient air, carbon dioxide (CO_2_), nitrogen (N_2_), and oxygen (O_2_) at a RH of 1%. The stability of switched domains varied significantly with atmospheric composition, with domains switched in CO_2_ being the most stable. Notably, we observed enhanced stability in an O_2_‐rich environment (1% RH) compared to ambient conditions (45% RH) and an O_2_‐rich environment at 38% RH, revealing that ferroelectricity in HZO is not absent in an O_2_‐rich environment. This work provides crucial insights into the role of environmental conditions in detecting ferroic response, offering a pathway to improved retention characteristics for memory applications.

## 2 Results and Discussion

### 2.1 Structure and Composition

**Figure 1:**
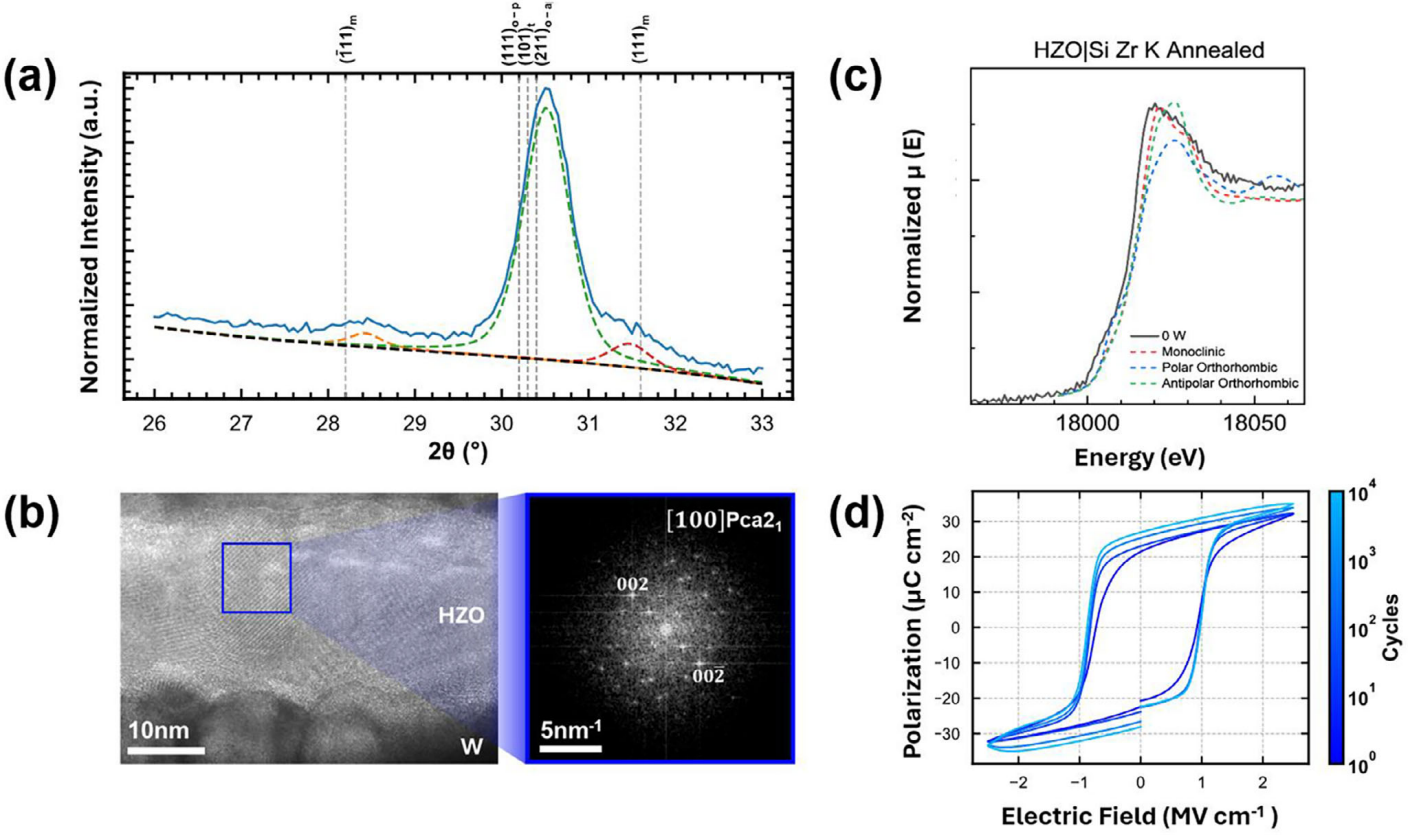
Structural and electrical characterization of the annealed HZO film. a) GIXRD pattern of HZO. The fits for each observed ${\left(\bar{1}11\right)}_{m}$ (orange dashed line), superimposed (111)_o‐p_/(101)_t_/(211)_o‐ap_ (green dashed line), and (111)_m_ (red dashed line) The peak is plotted below each corresponding pattern. The grey dashed lines represent nominal peak positions for the various phases of HZO, b) HRTEM with FFT displaying *Pca*2_1_ phase imaged along a [100] zone axis, c) XANES spectra of the Zr K edge, along with FEFF simulated standards, and d) P‐E hysteresis loops of HZO films. In (a), m refers to monoclinic, o‐p refers to orthorhombic polar phase, o‐ap refers to anti‐polar orthorhombic phase, and t refers to tetragonal phase.

The GIXRD pattern of the annealed HZO film after etching the top tungsten layer is shown in **Figure** **1**a. The results confirm the presence of metastable phases. However, due to the structural similarity between the orthorhombic and tetragonal phases of hafnia, the primary diffraction peak can be attributed to the (111) polar orthorhombic phase (o‐p), the (101) tetragonal phase (t), and the (211) anti‐polar orthorhombic phase (o‐ap). Notably, no peaks associated with the equilibrium monoclinic phase (m) were observed in films with an intact top tungsten layer (Figure S4, Supporting Information). However, after the removal of the tungsten layer, approximately 10 vol.% of the monoclinic phase was detected, in agreement with previous reports.^[^ ^32^ ^]^ To quantify the relative fractions of metastable and monoclinic phases, the intensity of each peak was integrated following the approach detailed in prior work.^[^ ^32^ ^]^ Further differentiation of metastable phases was achieved through FTIR studies, as shown in Figure S5 (Supporting Information). The FTIR spectrum exhibits distinct absorbance peaks at ≈700 and ≈770 cm^−1^, which are characteristic of the *Pca*2_1_ phase in hafnia, consistent with prior studies.^[^ ^33^, ^34^ ^]^

High‐resolution transmission electron microscopy (HRTEM) studies verified the presence of the *Pca*2_1_ phase, as shown in Figure 1b. The fast Fourier transformation (FFT) displays a grain imaged along a [100] *Pca*2_1_ zone axis. Energy dispersive X‐ray spectroscopy confirmed the stoichiometric ratio of Hf and Zr atoms in these HZO thin films (see Figure S6, Supporting Information). The actual composition of the prepared films, as measured with STEM‐EDS, was found to be Hf_0.56_Zr_0.44_O_2_, which is in good agreement with the targeted 6:4 (Hf:Zr) growth ratio. To get further insights into the structural properties, XANES studies were also carried out. Figure 1c presents the XANES spectra for the Zr K edge of the HZO thin film alongside FEFF‐simulated polymorphs. The Zr K edge, shown in Figure 1c, corresponds to 1s → 5d transitions. The two features present in the Zr K edge indicate the presence of the polar orthorhombic phase based on the FEFF calculations (the pre‐edge peak and the post‐edge shoulder, both denoted by arrows). The Hf L_3_ and Hf L_1_ XANES spectra are given in Figure S7 (Supporting Information), where the same fingerprinting technique was applied and further solidifies the presence of the polar orthorhombic phase.

### 2.2 P‐E Hysteresis Measurements

Polarization–electric field (P‐E) hysteresis loops as a function of cycling are shown in Figure 1d. A clear hysteretic response was observed for the first cycle. PUND responses taken at each cycling interval versus electric field are given in Figure S8 (Supporting Information). No significant variations in remanent polarization or coercive field were observed over cycling from 10^1^ to 10^4^ cycles. The extracted values of remanent polarization and coercive field were 22 µC cm^−2^ and 1 MV cm^−1^, respectively. The voltage across a 20.2 nm thick layer with an electric field of 1 MV cm^−1^ is approximately 2.02 V.

### 2.3 PFM Results

#### 2.3.1 Role of Humidity on Ferroelectric Behavior of HZO Films

**Figure 2:**
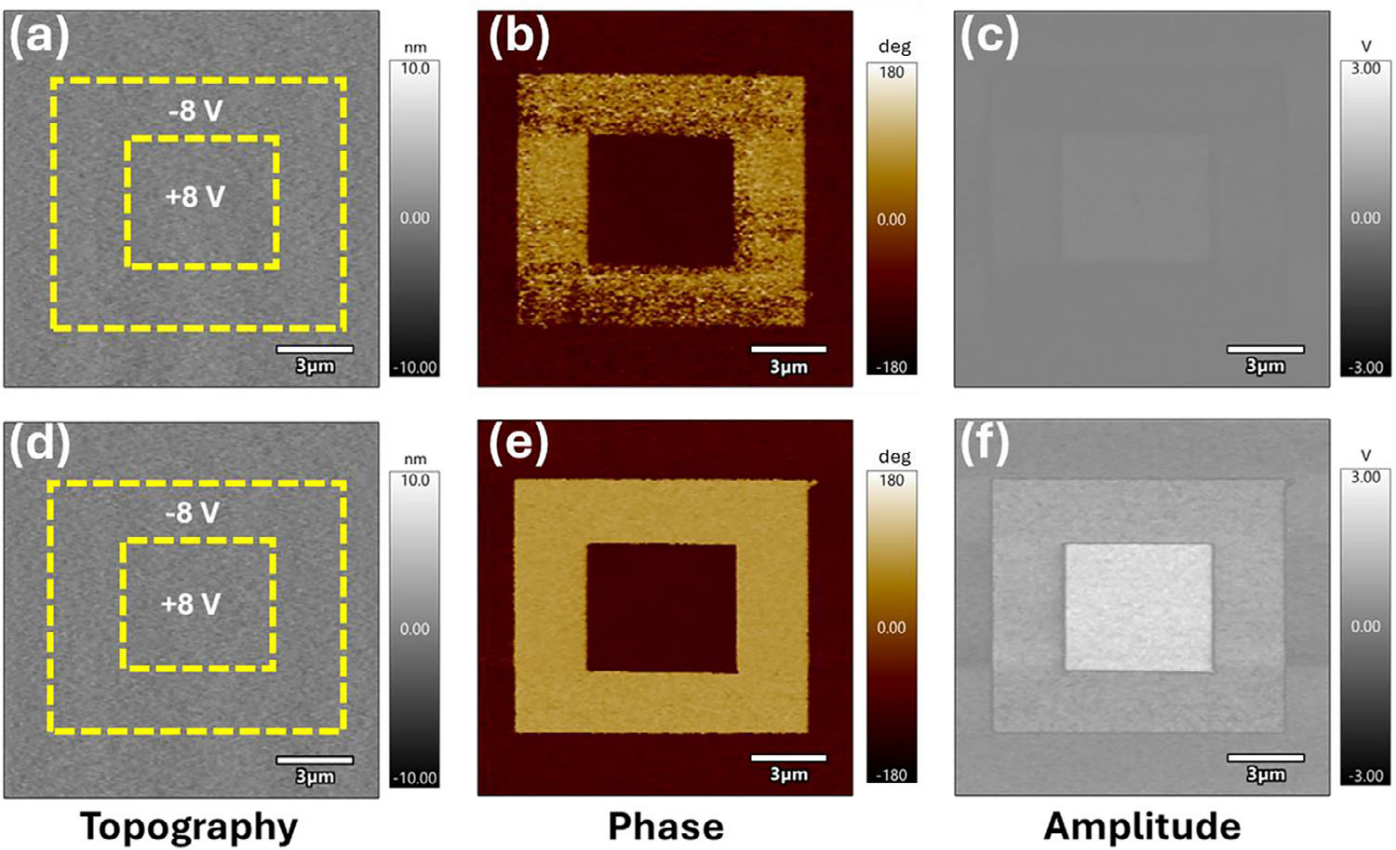
PFM measurements in different humidities. a) Topography and PFM b) phase and c) amplitude images in ambient conditions (RH = 45%) after surface treatment. d) Topography and PFM e) phase and f) amplitude images in a low‐humidity N_2_ environment (RH = 1%).

We first examined the PFM phase and amplitude response of HZO films alongside a typical ferroelectric material, BiFeO_3_ (BFO), and a non‐ferroelectric thin film material, amorphous Al_2_O_3_, under ambient conditions (RH = 45%). This was done to verify whether the PFM response of HZO films is a genuine ferroelectric response or arises from electrostatic origins. BFO films showed clear and well‐defined domains, while these features were absent in both the HZO and Al_2_O_3_ samples (Figure S9, Supporting Information). Classical box‐in‐a‐box poling experiments were then performed on these films under the same conditions, applying a DC bias of ‐8 V to 10 µm x 10 µm areas and +8 V to 5 x 5 µm areas. For the BFO film, well‐defined polarization switching occurred within the poled regions (Figure S10, Supporting Information). However, poling was not observed in the HZO and Al_2_O_3_ films. Interestingly, after a surface treatment, which involved cleaning with alcohol and water, followed by nitrogen blow (Text S1, Supporting Information), the polarization switching became possible for the HZO film. The topography and PFM amplitude and phase images after poling the HZO sample with a DC bias of ± 8 V are shown in **Figure** **2**a–c. No change in surface topography was observed after applying the DC bias, and the surface roughness was measured to be 0.7 ± 0.1 nm, indicating the high quality of the films. The PFM phase showed a well‐defined 180° contrast. The PFM amplitude images revealed an asymmetric contrast between regions polarized by positive and negative bias. The unpoled region outside of the yellow box shared the same phase with +8 V poling, indicating a preferred downward polarization of the films. However, the regions poled with a positive voltage exhibited a higher amplitude compared to those poled with a negative voltage. Notably, the negatively poled domains were unstable, as evidenced by a continuous reduction in contrast in both phase and amplitude images that eventually disappeared after ∼15 minutes. Balke et al. showed a similar trend in ultrathin films of lead zirconate titanate, where the bottom electrode and other intrinsic defects were modelled as driving factors for backswitching.^[^ ^35^ ^]^ However, a recent investigation into HZO films grown by atomic layer deposition on several substrates demonstrated that the asymmetry of hysteresis loops, and therefore the stability, is governed by film thickness rather than substrate type.^[^ ^29^ ^]^ Therefore, the influence of the bottom electrode can be neglected. Moreover, compared to P‐E measurements, shown in Figure 1c, significantly higher switching voltages (more than four times the coercive field) were required to achieve 180° ferroelectric domain switching using PFM (Figure 2a–c). This suggests that the domain switching behavior and retention properties may be predominantly influenced by intrinsic defects or interactions between the film surface and the ambient atmosphere.

To investigate the role of atmospheric interactions, we performed a similar experiment in an N_2_ environment at a RH of 1%, as shown in Figure 2d–f. Both the PFM phase and amplitude exhibited similar trends to those in ambient, but with a few notable differences. First, the phase image revealed a clear 180° contrast, and the amplitude image showed sharply defined boundaries, unlike those observed under ambient conditions; second, the amplitude signal was much higher in the N_2_ environment; and finally, the up‐polarized domains remained stable for several hours. The reduction in PFM amplitude in ambient compared to low RH conditions was also observed during single‐point contact resonance frequency sweeps on HZO; however, an opposite trend was observed for BFO (Figure S11, Supporting Information). For HZO, the frequency sweeps were also recorded as a function of loading force, and the PFM amplitude was always higher at low RH (Figure S12, Supporting Information). A detailed discussion about the amplitude variation in low and high humidity environments is given in Text S2 (Supporting Information). The observed ferroelectric behavior can be attributed to the substantial reduction in RH (by 98%) compared to ambient conditions. However, achieving low humidity required introducing dry N_2_ into the chamber, not only reducing moisture content but also altering the gaseous environment. The effect of this environmental change will be addressed in subsequent sections.

Modification of PFM amplitude from HZO films has been reported before. Kim et al. reported an enhanced amplitude in HZO films subjected to He ion irradiation that increased the amount of oxygen vacancies.^[^ ^36^ ^]^ In the case of the N_2_ environment, the amplitude enhancement we observed might be attributable to the creation of new oxygen vacancies or the retention of existing ones; however, the creation of new oxygen vacancies is less probable, as even with the application of a lower AC voltage, a higher amplitude signal was observed. Therefore, the preservation of oxygen vacancies appears to be the more plausible factor in the N_2_ environment. Moreover, the complete PFM phase reversal (Figure 2e) indicates a high fraction of the ferroelectric orthorhombic phase in the films. There is no evidence of localized defect clusters or a non‐ferroelectric phase, which aligns with the XRD results (Figure 1a). For comparative analysis, BFO and Al_2_O_3_ samples were also tested in a N_2_ environment. No domain wall formation or PFM contrast was observed in the Al_2_O_3_ film. However, in the case of the BFO film, domain‐switching was only possible at larger voltages (±10 V) (Figure S13, Supporting Information). Given the increased PFM amplitude and demonstrated switching, it was hypothesized that HZO films could exhibit domain switching at lower poling voltages in low humidity conditions. The PFM phase and amplitude images with domains switched at varying voltages are shown in Figure S14 (Supporting Information); domains could be switched at just 2 V, which is comparable to values obtained from macroscale P‐E loop measurements (Figure 1d).

Recent investigations highlighting 180° phase contrast even in non‐ferroelectric samples due to electrostatic contributions underscore the limitations of PFM data, making such results less conclusive.^[^ ^37^, ^38^, ^39^, ^40^ ^]^ To address this, two control experiments were conducted to confirm that the observed PFM phase contrast arises from ferroelectric behavior rather than charge injection. In the first experiment, polarization domains were written in the HZO film using an AFM probe, and the response was measured by applying an AC voltage to the bottom electrode and then to the tip. Clear domains and domain walls were consistently observed in both configurations. If the contrast were solely due to charge injection, the response would have been either absent as injected charges penetrate only a few nanometers (≈5–10 nm) into the film or scattered due to the high dielectric constant of HZO. The observed results confirm that the phase contrast originates from ferroelectric behavior (Figure S15, Supporting Information). In the second experiment, we carried out the first and second‐harmonic AC voltage sweep measurements for BFO, HZO, and Al_2_O_3_ samples (Figure S16, Supporting Information). The first harmonic amplitude showed linear response and exceeded the second harmonic response in HZO and BFO, whereas the Al_2_O_3_ sample showed the opposite trend. This distinct behavior confirms^[^ ^41^ ^]^ the piezoelectric nature of HZO and BFO while underscoring the expected absence of piezoelectricity in Al_2_O_3_ (Text S3, Supporting Information). The piezoelectric coefficient (d_33_) for HZO in low‐humidity N_2_ conditions was found to be 1.8 ± 0.2 pm V^−1^ (Figure S17, Supporting Information). Overall, these findings provide further evidence that the PFM phase contrast in HZO is due to the intrinsic ferroelectric response.

#### 2.3.2 BEPS Measurements of HZO Films in Ambient Versus Controlled Environment

**Figure 3:**
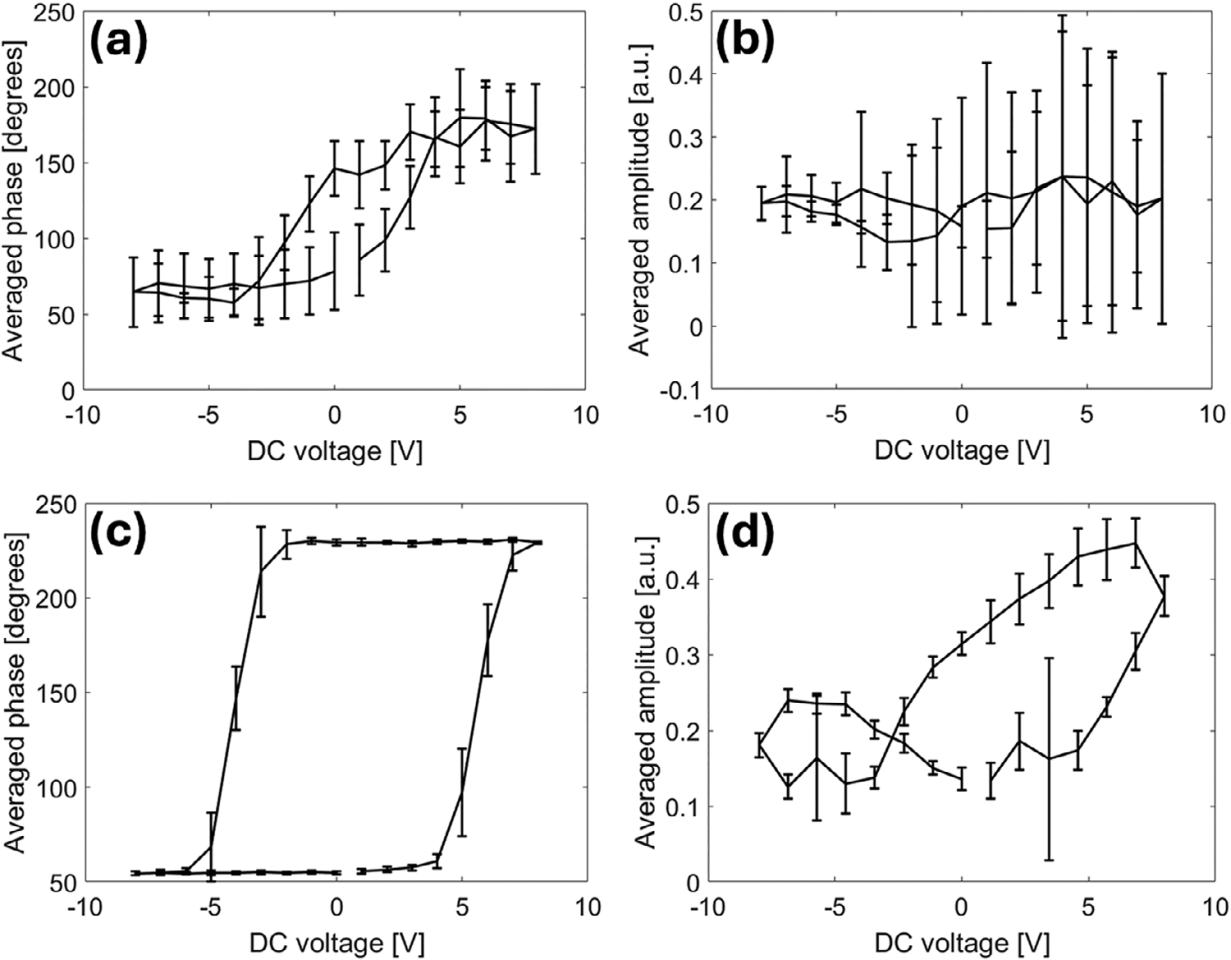
BEPS measurements in different humidities. BEPS a) phase and b) amplitude loops (average of 100 loops measured within a 5 × 5 µm area) of HZO in ambient (RH = 45%), and BEPS c) phase and d) amplitude loops of HZO in N_2_ environment (RH = 1%).

Following the observation of unconventional behavior in HZO films, BEPS data were collected in ambient and N_2_ environments, as shown in **Figure** **3**. In ambient conditions, the BEPS phase loop shows weak domain contrast and pinching of the loop, indicating non‐ferroelectric/antiferroelectric‐like behavior (Figure 3a). This is further supported by a weak and symmetric BEPS amplitude response, as shown in Figure 3b. In contrast, in an N_2_ atmosphere, the phase loop exhibited well‐defined 180° switching, consistent with ferroelectric switching. Moreover, the amplitude loop showed a clear asymmetry, consistent with the PFM imaging studies shown in Figure 2. These BEPS loops provide compelling evidence of an environment‐induced transition from non‐ferroelectric to ferroelectric behavior in these films. These findings are in line with the trends observed in UHV conditions, where the ferro‐ionic model of HZO has been taken into consideration.^[^ ^30^ ^]^ The environment‐induced transition from non‐ferroelectric to ferroelectric behavior highlights the crucial role of electrochemical coupling in inducing the polarization response in HZO films. In ambient conditions, the HZO films appear to have a surface‐mediated non‐ferroelectric response through oxygen vacancy passivation, while the controlled environments, such as N_2_, facilitate the ferroelectric stability by preserving the oxygen vacancies. This mechanism is consistent with measurements in capacitor geometries, highlighting the role of oxygen vacancy preservation in determining the ferroelectric response of hafnia‐based thin films.

#### 2.3.3 Role of Surrounding Gaseous Environment on Domain Relaxation of HZO Films

**Figure 4:**
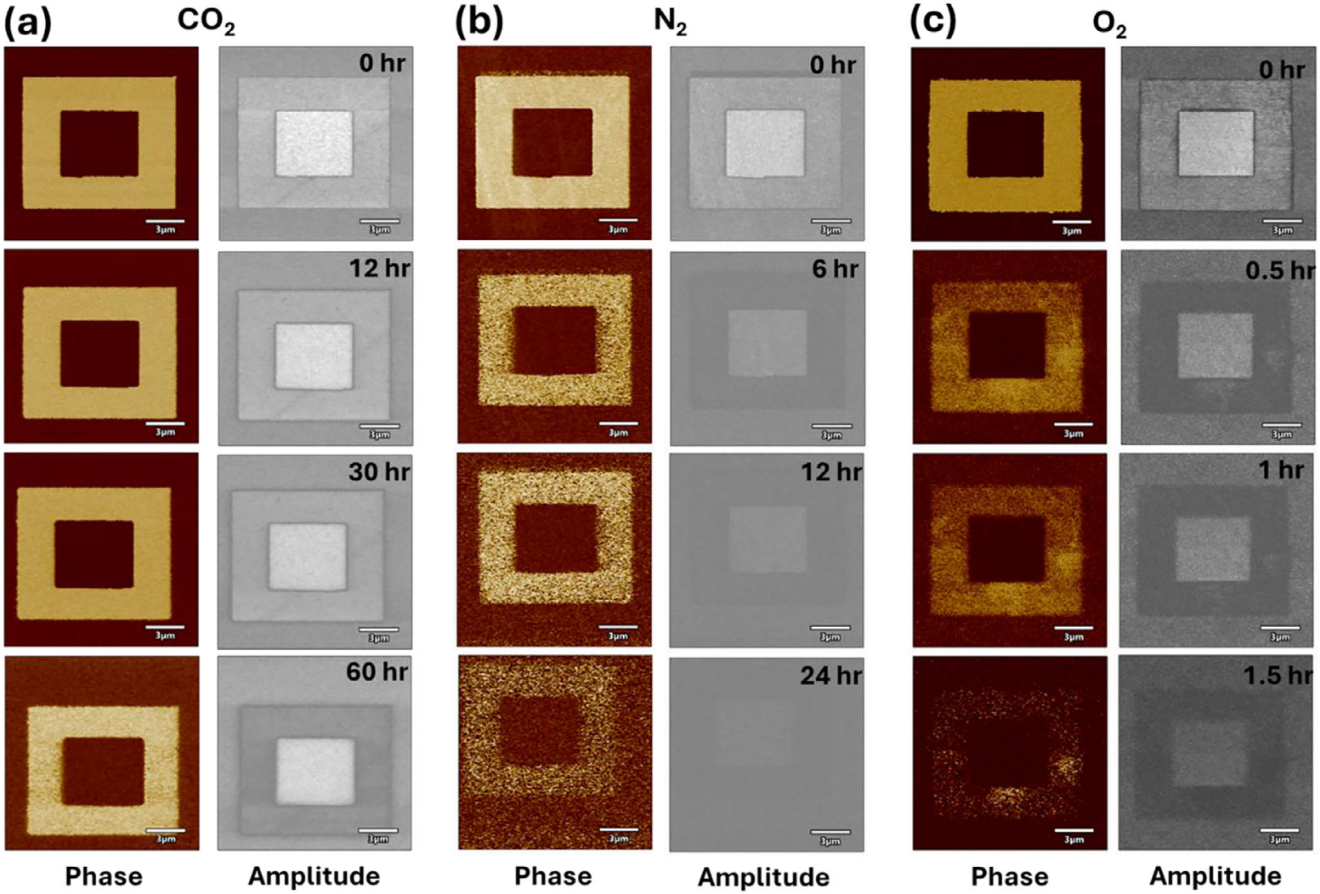
Stability of domain switching in different gases. PFM phase and amplitude contrast variation over time at 1% RH in a) CO_2_, b) N_2_, and c) O_2_ environments after switching with ±8 V (+8 V to central square). The amplitude scale is −3 to + 3 V in CO_2_ and N_2_ and −1.5 to + 1.5 V in O_2_. The phase scale is −180° to +180° V for all phase image panels.

Next, considering that written domains were longer lasting in N_2_ compared to ambient conditions, we investigated the influence of surrounding gases on polarization stability. These experiments were performed in different controlled environments (CO_2_, N_2_, and O_2_) while maintaining a constant humidity level (RH = 1%), set point (1 V), and AC voltage (0.5 V), and by using a new probe tip for each experiment. The domain stability over time under different ambient conditions is shown in **Figure** **4**. Examining the phase and amplitude contrasts, both decayed over time and eventually disappeared after varying intervals depending on the surrounding gases. The results revealed that the domains exhibited the highest stability in a CO_2_ environment, remaining stable for more than 60 h (115 h in another measurement, even at RH = 3%, with fewer scans (Figure S18, Supporting Information). This was followed by N_2_, where domains persisted for 20 h, and for 1.5 h in O_2,_ while in ambient air, lasting only for 15 min. Although humidity was maintained at 1% across all three environments, domain relaxation exhibited significant variation, indicating that the surrounding atmosphere plays a dominant role in governing the domain relaxation dynamics in these films. These results suggest that the controlled environment at the HZO surface is key to unlocking ferroelectricity in these films. Notably, the surface topography does not exhibit any change in any of these controlled environment conditions, indicating that there was no surface degradation. It is important to note that these scans were conducted at discrete intervals, with the same scan frequency for all environments. The number of scans impacted domain stability, providing additional insights into the role of scanning frequency in domain relaxation (Figure S19, Supporting Information).

Moreover, the amplitude variation of upward‐switched domains demonstrated a strong dependence on the surrounding gas. For downward‐switched domains (preferred orientation), the amplitude gradually decayed over time until it matched the virgin state values. In contrast, for up‐switched domains (which are inherently less stable), the amplitude followed a distinct trend across all three gaseous environments, initially decreasing to a level lower than the virgin state, then gradually increasing until it returned to its original value. This transition showed a strong dependence on both the surrounding gaseous environment and the initial poling voltage used for domain switching. The amplitude variation over time of these films in CO_2_ is given (Figure S20a, Supporting Information). In ambient, the amplitude signals decayed in the up‐polarized (negative DC bias) region, as shown in Figure 2, to below that of the virgin state. In contrast, in the other three environments (CO_2_, N_2_, and O_2_), the amplitude signal remained higher in the negatively biased region than in the virgin state and decreased over time. However, in the O_2_ environment, the amplitude signal dropped below the virgin state just after the second scan (5–10 min), while in N_2_, this occurred after 12 h, and in CO_2_, after 45 h. The significantly lower stability in O_2_ compared to N_2_ and CO_2_ suggests the passivation of oxygen vacancies leads to a rapid decay of domains.

However, the domain retention in O_2_ remained higher than in ambient conditions, indicating that other atmospheric constituents may inhibit the ferroic response in these films. This inhibition could result from passivation of oxygen vacancies by other atmospheric constituents or increased depolarization field induced by water adsorption on the HZO surface. Moreover, the amplitude transition showed a strong dependence on applied voltage to switch the domains, as shown in Figure S20b (Supporting Information). The amplitude decay in these films is primarily due to domain relaxation. As time progresses, the polarization within the ferroelectric material can decrease because of backswitching, which occurs through the nucleation and growth of reversed domains. This process leads to a reduction in the retained polarization, which directly affects the amplitude of the signal observed in the device.

#### 2.3.4 Combined Effect of Relative Humidity and Gaseous Environment on Domain Relaxation of HZO Films

Next, to specifically isolate the contribution of the water layer, we repeated the experiments under a humidified O_2_ environment (RH = 38%). Interestingly, while the HZO films exhibited successful switching with clear contrast, a higher voltage was required to switch domains in wet O_2_ compared to dry O_2_. Moreover, the domains relaxed more rapidly in wet environments than in dry environments (less than 60 min in wet O_2,_ see Figure S21a, Supporting Information). This indicates that the stability of these domains is severely compromised by the presence of polar water molecules in the atmosphere. A detailed comparison of switching voltages under dry and wet O_2_ conditions is provided in Figure S21b (Supporting Information). These findings are in line with a recent study by Wei et al., which demonstrated that increasing humidity reduces upward polarization retention.^[^ ^28^ ^]^ They attributed the observed backswitching to the built‐in electric field generated by water molecule adsorption on the film surface. However, our investigations reveal a dramatic variation in relaxation dynamics under different gaseous environments, even with constant humidity, suggesting that domain retention is not exclusively governed by humidity but arises from the cumulative interplay of multiple environmental factors. Moreover, the surface topography does not exhibit any change in any of these controlled environment conditions, indicating that there was no surface alteration resulting from the chemical environment or biased application. Furthermore, after writing the domains in a controlled atmosphere (CO_2_, N_2_, or O_2_), exposure of the film surface to ambient atmospheric conditions (by turning off the gas flow) resulted in the immediate decay of the switched domains, reverting to the preferred downward polarization state as water re‐adorbs to the surface (Figure S22, Supporting Information). The adsorbed water might provide dissociated ions, a backswitching field, and/or interact with near‐surface oxygen vacancies.

### 2.4 KPFM Results

**Figure 5:**
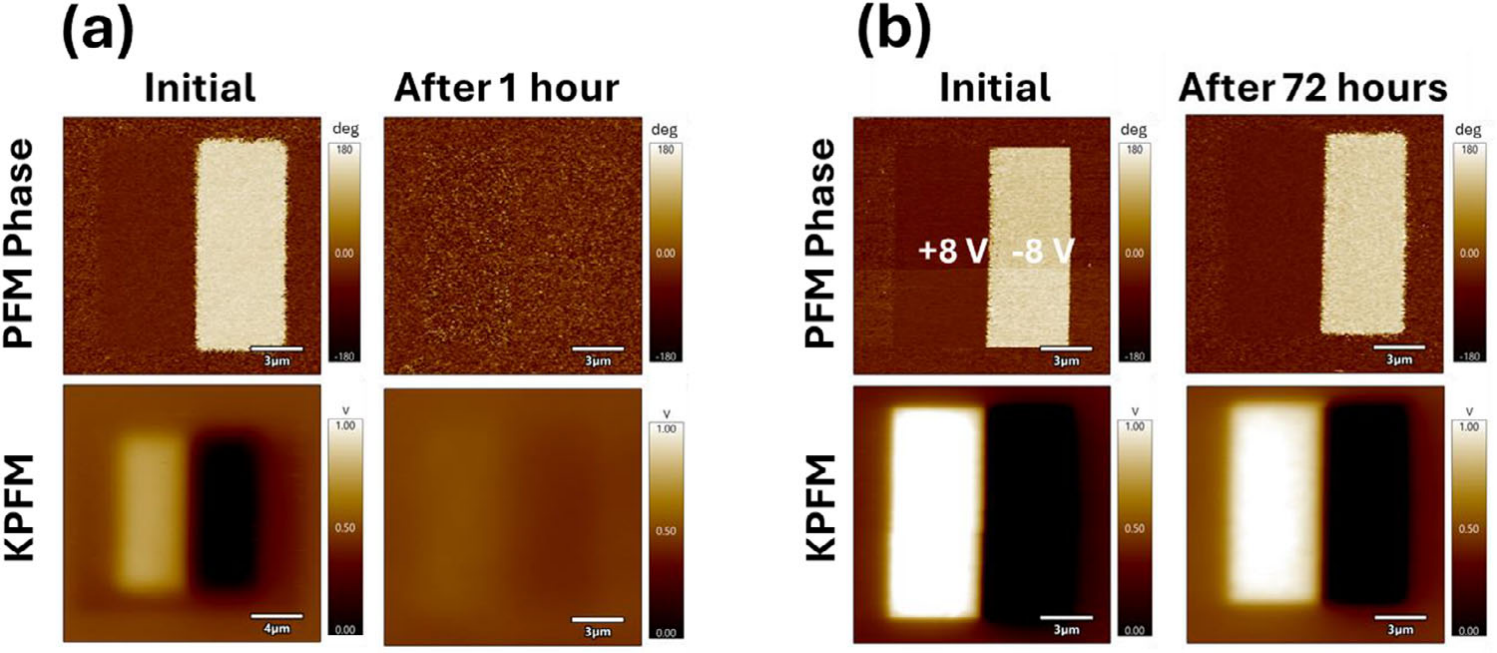
Correlated PFM and KPFM measurements. Comparison of PFM and KPFM contrast in a) ambient (45% RH) and b) controlled (CO_2_, 3% RH) environment.

KPFM was performed, along with PFM, to get further insights into the role of moisture on domain relaxation at different stages following the writing process in ambient conditions and in a controlled CO_2_, 3% RH environment. KPFM is responsive to several electrostatic contributions, including polarization, screening, and injected charge, and can be used to better understand what is happening when a ferroelectric domain is switched. Interestingly, KPFM contrast was lower in ambient conditions compared to controlled environments, and the PFM phase decay exhibited a direct correlation with variations in KPFM contrast. In ambient, the PFM phase contrast decayed within an hour, and the KPFM contrast was severely reduced (**Figure** **5**a), whereas in CO_2_, the PFM phase and KPFM contrast remained strong after 72 h (Figure 5b). Both KPFM and PFM contrasts are notably reduced in ambient compared to CO_2_. While the PFM images follow the writing pattern with high fidelity, the contact potential difference (CPD) signal in the KPFM images extends beyond the domain boundaries, especially in ambient conditions, highlighting that lift‐mode KPFM has reduced resolution compared to contact‐mode PFM, related to the long‐range nature of electrostatic forces and the role of screening by the water layer. The charge appears to spread out and dissipate in the ambient due to charge diffusion (Figure S23, Supporting Information). The CPD is positive when positive DC bias is applied and negative when negative DC bias is applied. The positive CPD appears to be more localized compared to the negative CPD, suggesting faster dissipation of negative charge and, consequently, lower retention of up‐polarized states. These findings are consistent with previous studies on the impact of RH on screening charge dissipation.^[^ ^42^ ^]^ Assuming the tip‐injected screening charge dominates the CPD signal, the reduced CPD contrast observed in ambient conditions compared to controlled environments is consistent with the model proposed by Wei et al., wherein a downward (irrespective of polarization direction) induced field due to adsorbed water acts to destabilize up‐polarized domains.^[^ ^28^ ^]^ Additionally, the more rapid dissipation of negatively injected screening charges may accelerate the decay of up‐polarized domains, potentially contributing to the reduced stability under high humidity levels.

### 2.5 Proposed Model

Our results establish that the domain stability of ferroelectric HZO films is highly sensitive to the chemical environment and measurement parameters. The films exhibited non‐ferroelectric‐like behavior in ambient conditions but showed a ferroelectric response under controlled environments. Domain relaxation was strongly dependent on the surrounding atmosphere and measurement factors, such as scan number and loading force. In this context, we propose a model to explain these observations.

**Figure 6:**
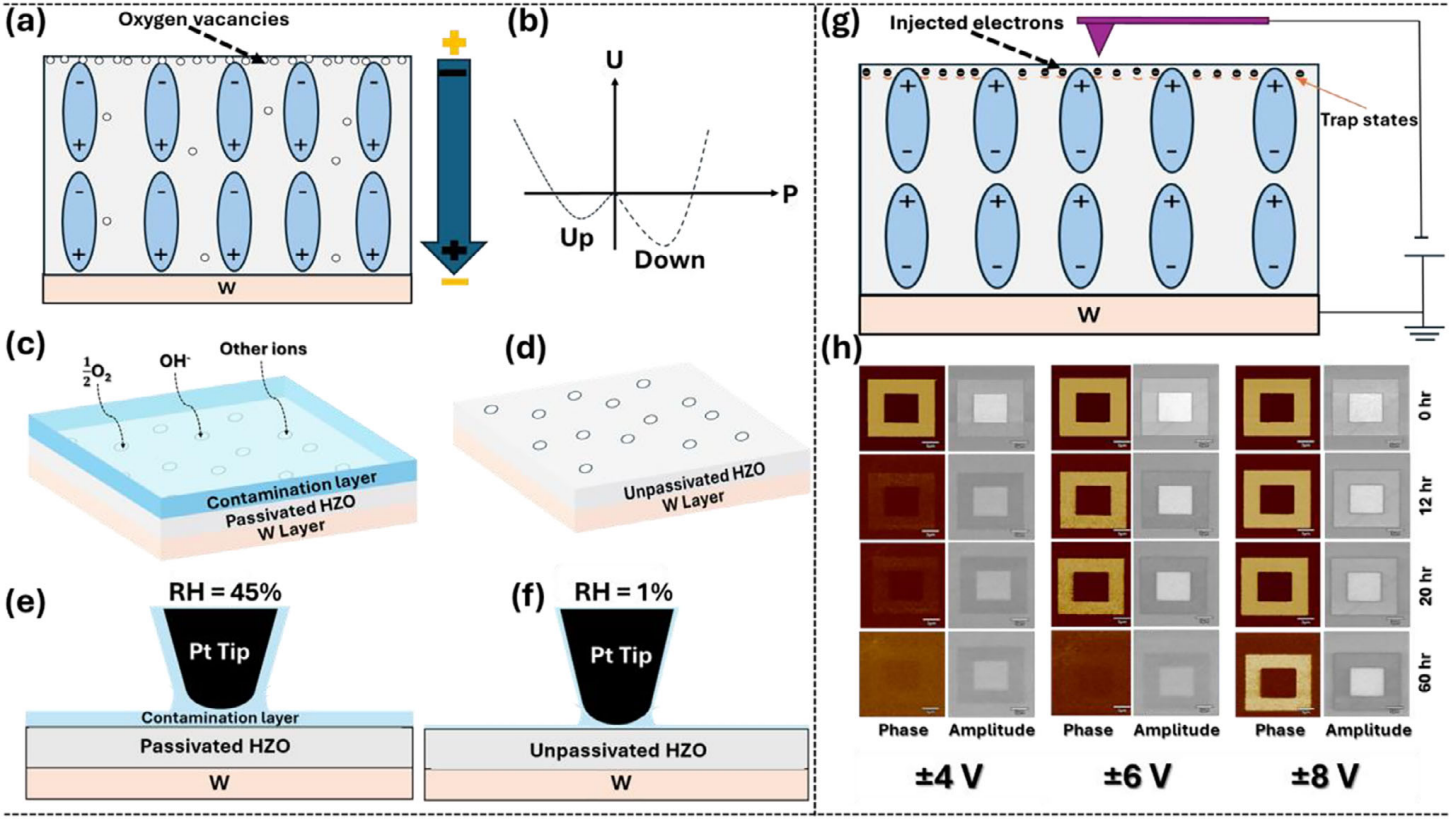
Model showing the interplay of oxygen vacancies, water, and injected charge. Schematics depicting a) the spontaneous polarization of the as‐prepared HZO films, where the bound polarization charges are screened by the electrode and oxygen vacancies (yellow ± signs represent screening charges), b) the energy diagram showing asymmetric polarization states, c) the HZO surface in ambient conditions, d) the HZO surface in a controlled environment, e) the water layer in ambient conditions, f) the water layer at low humidity, g) injected electrons upon polarization reversal, and h) switching stability as a function of switching voltage.

**Figure** **6**a illustrates the initial downward polarization in HZO thin films. In their as‐prepared state, the spontaneous polarization aligns downward toward the tungsten bottom electrode. It is likely that the positively charged oxygen vacancies at the top surface (they can also exist elsewhere) aid in screening the polarization (Text S4, Supporting Information).^[^ ^43^ ^]^ As a result, the downward polarization state is preferred, as shown in Figure 6b and supported by PFM and BEPS data.

It is well established that these oxygen vacancies play a key role in stabilizing the metastable orthorhombic (*Pca*2_1_) phase responsible for ferroelectricity in HZO.^[^ ^17^, ^36^, ^44^, ^45^ ^]^ Figure 6c depicts the proposed behavior of the film in ambient conditions. In these conditions, when the film is exposed to the atmosphere, the surface interacts with environmental species like OH^−^, O_2_, or other constituent ions. These physiosorbed species potentially passivate oxygen vacancies, altering the surface composition and resulting in non‐ferroelectric behavior. In controlled environments like N_2_, O_2_, or CO_2_, the concentrations of surface reactive species reduce drastically, as depicted in Figure 6d. As a result, as‐prepared oxygen vacancies are preserved, facilitating ferroelectric switching. To support this hypothesis, we carried out XPS analysis on HZO films that were pre‐treated under different atmospheric conditions to examine their surface chemistry and oxidation behavior. The detailed methodology is provided in Text S5 (Supporting Information). The samples were briefly exposed to ambient air (for approximately 5 min) during the transfer process from the gas‐treated environment to the XPS system via sample mounting. While reversion from a less ferroelectric to a more ferroelectric state upon atmospheric exposure can occur rapidly (as shown in Figure S22, Supporting Information), the XPS data revealed clear and consistent differences in surface chemistry across the different gas treatments. Core‐level spectra for O 1s, Hf 4f, and Zr 3d are presented in Figure S25 (Supporting Information). These results indicate that oxidation of Hf and Zr ions and hence the oxygen bonding environments vary depending on the ambient gas. Under O_2_ treatment, both O:Hf and O:Zr ratios are highest, and the binding energies of Hf 4f_7_/_2_ (≈17.45 eV) and Zr 3d_5_/_2_ (≈182.95 eV) are also elevated, indicating enhanced oxidation (i.e., likely passivation of oxygen vacancies). In contrast, the N_2‐_treated sample shows the lowest atomic ratios and binding energies, suggesting lower oxidation of metal ions and hence, a higher concentration of oxygen vacancies. CO_2_ treatment results in an intermediate level of oxidation and binding energy. These observations highlight the critical role of atmospheric exposure in determining the oxidation states of Hf and Zr, which can in turn influence the ferroelectric properties and defect landscape of the HZO films. Our hypothesis that O_2_ treatment effectively passivates oxygen vacancies, thereby accelerating the back‐transition to a less ferroelectric state, is well supported by the significantly higher oxidation levels observed in the XPS data under O_2_ conditions. Text S6 (Supporting Information) presents a detailed discussion about the core‐level spectra of Hf 4f, Zr 3d, and O 1s, along with their corresponding quantitative ratios.

Furthermore, the higher coercive field and reduced stability in high humidity conditions can also be attributed to adsorbed water on the HZO surface. These adsorbed molecules cause an electric field directed toward the bottom electrode. This field destabilizes the upward switched domain. A detailed analysis of the depolarization field due to adsorbed water molecules is provided in the reference.^[^ ^28^ ^]^ In addition to the depolarizing field, the acidic nature of atmospheric moisture may also contribute to domain relaxation; however, this aspect falls beyond the scope of the present study. Notably, the adhesion force is reduced by 88% in controlled environments (from 123 ± 3 to 15 ± 2 nN), while changing the humidity from 45% to 1% (Figure S24, Supporting Information). Under RH conditions of 1%, water typically forms around two molecular layers and exhibits highly structured ice‐like behavior, as depicted in Figure 6f.^[^ ^42^, ^46^ ^]^ This structured state reduces the likelihood of water dissociation into H^+^ and OH^−^ ions, suggesting that passivation or screening in such environments is minimal.^[^ ^42^, ^46^ ^]^ Conversely, at higher humidity levels, the dissociation of water molecules into ions on ferroelectric surfaces becomes more pronounced, a phenomenon that has been extensively explored in the literature.^[^ ^24^, ^25^, ^26^, ^42^, ^46^, ^47^ ^]^ This explains why, at humidity levels of ≈1%, the thin water layer fails to passivate the oxygen vacancies, and the ferroelectric properties of HZO films remain intact.

Now, moving on to the next aspect of gas‐dependent stability, where domains written in CO_2_ are more stable than those written in N_2_, which are more stable than those in O_2_, which are more stable than those in ambient. The comparatively higher stability in O_2_ versus ambient can be attributed to lower humidity. However, the reduced stability in O_2_ in comparison to CO_2_ and N_2_ is due to partial passivation of oxygen vacancies by physiosorbed O_2_ as revealed by XPS investigations. Most of these vacancies appear to become passivated in ≈5 –10 min of scanning time. Comparing N_2_ and CO_2_, N_2_ is linear and less polarizable (strong triple bond), due to its small, tightly bound electron cloud. However, it has been reported that partial positive and negative charges can develop on carbon and oxygen atoms of CO_2_, because it has a larger and more diffuse electron cloud, making it more easily distorted by external electric fields.^[^ ^48^, ^49^ ^]^ Preliminary XPS results also provide indirect evidence supporting the differing behavior of our films when treated in CO_2_ versus N_2_ atmospheres, as discussed in Figure S25 (Supporting Information). From these investigations, it appears that CO_2_ binds more strongly to the surface than N_2_. However, to fully elucidate the behavior of hafnia films in various gaseous environments, further investigations are required.

Repeated scanning has been reported to remove adsorbed charge, so even though CO_2_ is more tightly attached to the surface, the more often the surface is scanned with the tip, the more likely the CO_2_ molecules can be dislodged.^[^ ^50^ ^]^ As well, we must consider the tip‐sample water meniscus. The water layer has not been completely removed, and the act of scanning amounts to sweeping the surface with a water meniscus that acts to increase the probability that ions or other atmospheric species in the water can passivate the near‐surface oxygen vacancies. Cumulatively, this has the unintended effect that the more we look at the stability of the switched domain, the faster the switched state decays.

To further understand the role of imaging measurement parameters and screening mechanisms in controlled environments, we considered a charge injection model. As is widely recognized, the availability of screening charges is considerably reduced in controlled low‐humidity or UHV environments compared to ambient conditions.^[^ ^30^ ^]^ In these conditions, screening is primarily governed either by intrinsic defects or charges injected into the film. During PFM, when the voltage applied to the tip exceeds the coercive field, it not only triggers polarization reversal but also injects charges into the ferroelectric surface.^[^ ^51^, ^52^ ^]^ For example, downward band bending at the surface of an up‐polarized domain would be favorable to the accumulation of electrons. Moreover, as discussed earlier, KPFM exhibited higher contrast in controlled environments compared to ambient conditions under the same applied voltage. Additionally, the decay of the PFM phase showed a direct correlation with variations in KPFM contrast. This suggests a correlation between domain stability, KPFM contrast, and oxygen vacancies. Oxygen vacancies create trap states that capture excess charge, thereby contributing to the stabilization of upward‐switched polarization, as shown in Figure 6g. Interestingly, the domain stability also showed a strong dependence on the applied DC bias for poling. Domains switched at ±8 V, ±6 V, and ±4 V in CO_2_ are shown in Figure 6h. Higher DC biases were observed to significantly enhance domain stability in CO_2_, with domains written at ±8 V, ±6 V, and ±4 V remaining stable for approximately 60, 20, and 12 h, respectively. This trend suggests that increasing the applied bias results in more injected charge, leading to prolonged screening (Figure S26, Supporting Information).^[^ ^53^ ^]^ The extended stability arising from the availability of more screening charges underscores the role of bias‐dependent charge injection in influencing domain retention. In addition, the variation in domain stability under continuous versus discrete scanning can be explained using the same screening model. Recent studies indicate that during contact scanning, the tip can remove surface adsorbates and charges, while also mechanically inducing polarization switching at higher loading forces, even without an applied bias.^[^ ^50^, ^54^ ^]^ Consequently, with more scans, surface screening charges dissipate more rapidly through the tip (when grounded), leading to faster domain relaxation. In cases where the circuit is left open, domain relaxation at higher loading forces occurs due to mechanical effects. Moreover, the reduced stability in high RH conditions or when exposed to ambient conditions (see Figure 5; Figure S22, Supporting Information) could also be due to the dissipation of these injected screening charges.

Our findings emphasize the critical role of controlling the chemical environment and measurement parameters in determining domain relaxation in hafnia‐based ferroelectrics using PFM. These insights open new possibilities for local material modification through the regulation of surface electrochemical states and tip‐mediated screening mechanisms to overcome technologically relevant issues for specific applications.

## 3 Summary

In summary, our study demonstrates a strong dependence of ferroelectricity in hafnia on the external chemical environment. The electrochemical state of the surface plays a crucial role in the stability of switched domains. While electrode‐capped films are ferroelectric in ambient, typical ferroelectric features were suppressed in bare films due to their ferroic‐ionic nature. However, treating hafnia‐based films with CO_2_, N_2_, or O_2_, particularly in low‐humidity environments, restored distinct ferroelectric behavior, with CO_2_ providing the highest domain stability. In these controlled environments, the surface interface behaves like a localized electrode, and the polarization screening and stability are enhanced by injected charge. Our findings offer a compelling explanation for the significant disagreements within the scientific community regarding the ferroelectric behavior of hafnia‐based films, highlighting the role of varying humidity levels and atmospheric partial pressures across different geographical locations alongside film quality as a key factor contributing to these inconsistencies.

## 4 Experimental Section

### 4.1 Film Deposition

The 20.2 nm‐thick HZO film was deposited using plasma‐enhanced atomic layer deposition at 260 °C in an Oxford FlexAL II system. The deposition was performed on a 100 nm tungsten bottom electrode, which was fabricated on a (001)‐oriented silicon substrate with a native oxide layer.^[^ ^55^ ^]^ Supercycles consisting of six cycles of HfO_2_ and four cycles of ZrO_2_ were used for HZO deposition. Precursors were tetrakis (ethylmethylamido) hafnium, tetrakis (ethylmethylamido) zirconium, and an oxygen plasma. Cation precursor doses of 1 and 1.5 s were used for HfO_2_ and ZrO_2_ layers, respectively. A 90 s purge followed each cation precursor dose. The oxygen plasma following each HfO_2_ layer had a power of 250 W and a duration of 3 s, while each ZrO_2_ layer used 300 W and 6 s. After each HfO_2_ deposition, the substrate was subjected to an atomic layer annealing step,^[^ ^56^ ^]^ which consisted of applying a 0 W bias using an auxiliary RF power supply to the substrate along with the use of a 250 W argon plasma for 5 s, as shown in Figure S1 (Supporting Information). A planar 20 nm thick tungsten top electrode was then sputtered on top, and the sample was annealed at 600 C for 30 s in a dynamic, 1 atm nitrogen environment using an Allwin21 AccuThermo AW 610 rapid thermal processor.

For macroscopic electrical characterization, a 50 nm thick platinum hard mask was sputtered on top through a shadow mask after annealing, and the uncovered tungsten was then etched with a tungsten etchant solution (K_3_[Fe(CN)_6_] and KOH) to create capacitor devices. A diagram of the HZO stacks after etching is shown in Figure S2 (Supporting Information).

### 4.2 X‐ray Reflectivity

The thickness of the HZO film was measured using X‐ray reflectivity with a Rigaku SmartLab diffractometer with a Cu Kα source. The resultant pattern was fit using SmartLab Studio II software. Both the resultant pattern and the theoretical fit are plotted in Figure S3 (Supporting Information).

### 4.3 Grazing‐incidence X‐ray Diffraction

HZO film phases were characterized using grazing‐incidence X‐ray diffraction (GIXRD) over a 2θ range of 26° to 33° with a fixed incidence angle ω of 1°. LIPRAS software was used to fit the patterns using Pseudo‐Voigt peak shapes.^[^ ^57^ ^]^

### 4.4 Fourier Transform Infrared Spectroscopy

Fourier transform infrared spectroscopy (FTIR) was performed using a Bruker INVENIO‐S instrument with a single‐reflection attenuated total reflectance attachment and DLaTGS detector.

### 4.5 X‐ray Absorption Fine Structure

X‐ray absorption near‐edge structure (XANES) measurements were performed at beamline 10‐ID at Argonne National Laboratory in Lemont, IL. The absorption spectrum of the Zr K, Hf L_3_, and Hf L_1_ edges of the HZO thin film was collected in fluorescence mode using a Lytle detector. Since the film was grown on a crystallized silicon substrate, the film was mounted on a spinner stage for measurement to mitigate Si diffraction peaks. The spectra were processed and analyzed using the Demeter package for X‐ray absorption spectra.^[^ ^58^ ^]^ FEFF9^[^ ^59^ ^]^ XANES calculations of the monoclinic, polar orthorhombic, and antipolar orthorhombic phases of HZO were used to provide insights into the expected electronic structure for the respective polymorphs. The FEFF calculations utilized a Hedin‐Lundqvist self‐energy, a full multiple scattering value of ≈11, and a self‐consistent field value of ≈5 for each polymorph simulated. Once convergence was reached, the simulated spectra were shifted in energy to match the experimental spectra.

### 4.6 Polarization and positive Up Negative Down Measurements

Polarization and positive up negative down (PUND) measurements were performed on pristine 100 µm diameter contacts using a Radiant Technologies Precision LC II ferroelectric tester. To measure the polarization response, 2.5 MV cm^−1^ maximum fields were applied with a period of 10 ms. PUND measurements were performed at 2.5 MV cm^−1^ field, 100 ms pulse delay, and 1 ms pulse width. Devices were field cycled using 2 MV cm^−1^ square waves with 1 kHz frequency.

### 4.7 Transmission Electron Microscopy

High‐resolution transmission electron microscopy (HRTEM) and scanning transmission electron microscopy (STEM) measurements were acquired on a ThermoFisher Scientific Talos F200X with a Super‐X energy‐dispersive X‐ray spectrometer operated at 200 kV. A Tescan focused ion beam (FIB) – secondary electron microscope Lyra 3 was used to fabricate the TEM specimen. FIB milling was performed with gallium ions at accelerating voltages of 30 kV (rough milling) and 5, 2, and 1 kV (fine polishing). Moreover, the compositional studies were carried out using STEM combined with Energy‑Dispersive Spectrometry (STEM‑EDS).

### 4.8 PFM Measurements

PFM measurements were conducted using an MFP‐3D AFM (Asylum Research) with Pt‐coated PPP‐EFM probes (Nanosensors). The nominal tip radius of the PPP‐EFM probes used throughout the study was around 25 nm. Typical values for the deflection sensitivity and spring constant for the probes used were ≈90–110 nm V^−1^ and ≈2.0–2.7 N m^−1^, respectively. Ferroelectric switching was induced by applying a bias voltage to the tip via an external lock‐in amplifier (Zurich Instruments HF2). All the measurements were carried out close to the contact resonance under consistent conditions (such as 0.5 V AC voltage was used for PFM imaging for all experiments), ensuring that the amplitude was comparable across all samples. Furthermore, for Kelvin Probe Force Microscopy (KPFM) measurements, a lift height of 50 nm was maintained. The scanning rate for PFM imaging was set to 1 Hz, while for KPFM, the scanning rate was maintained at 0.5 Hz. Band excitation (BE)‐PFM data was obtained by integrating AFM measurements with a National Instruments fast data acquisition card (NI PXI‐6115). Custom software generated probing signals and recorded local BE piezoresponse spectroscopy (BEPS). BEPS measurements were performed at frequencies between 300–400 kHz. Furthermore, the PFM/KPFM measurements in controlled environments were carried out using a fluid cell (MFP‐3D, Asylum Research) to maintain a controlled gas environment and desired RH using a humidity sensor (HIH4000, Honeywell, ±3.5% accuracy). Other than in ambient, measurements were carried out in N_2_, CO_2_, and O_2_ environments with gases flowing at ≈0.1 bar (at a rate of ≈0.5 L min^−1^n for O_2_) to control the RH inside the cell. Prior to the SPM measurements, the samples were cleaned (Text S1, Supporting Information) and kept in the respective gas environments for 6 hours.

### 4.9 X‐ray Photoelectron Spectroscopy Measurements

X‐ray photoelectron spectroscopy (XPS) spectra were acquired using a Scienta Omicron XM1200 instrument equipped with a monochromated Al Kα source (1486.7 eV) and a pass energy of 50 eV to analyze the chemical composition of the HZO samples. The XPS data were analyzed and fitted using Aanalyzer software, and the spectra were plotted using OriginPro 7.5. The sensitivity factors used for quantification were 2.221 for Hf 4f, 2.216 for Zr 3d, and 0.711 for O 1s.

## Conflict of Interest

The authors declare that they have no conflicts of interest related to this work.

## Supporting information



Supporting Information

Supplementary Data

## Data Availability

The data that support the findings of this study are available from the corresponding author upon reasonable request.
