# Supplementary material for: Environmental Control of Ferroelectricity in Hafnia Films
Source: Adv Mater. 2025 Aug 7;37(42):e03852. doi: 10.1002/adma.202503852 (PMC12548525; doi:10.1002/adma.202503852)
Supplement: Supplementary file 1 — Supporting Information [file ADMA-37-e03852-s002.docx]

**Supporting Information**

**Environmental Control of Ferroelectricity in Hafnia Films**

Waseem Ahmad Wani^1^, Nicolas K. Lam^2^, Kristina Holsgrove^3^, Gerald Bejger^4^, Tinsae Alem^2^, Kory Burns^2^, Stephen J. McDonnell^2^, Christina M. Rost^4^, Amit Kumar^3^, Jon F. Ihlefeld^2,5^, Brian J. Rodriguez^1*^

^1^School of Physics and Conway Institute, University College Dublin, Belfield, Dublin 4, Ireland

^2^Department of Materials Science and Engineering, University of Virginia, Charlottesville, Virginia 22904, USA

^3^School of Mathematics and Physics, Queen's University Belfast, Belfast, UK

^4^Department of Materials Science and Engineering, Virginia Polytechnic Institute and State University, Blacksburg, VA 24061, USA

^5^Charles L. Brown Department of Electrical and Computer Engineering, University of Virginia, Charlottesville, Virginia 22904, USA

**Supplementary Table 1: Switching voltage characteristics of Hf_0.5_Zr_0.5_O_2_ (HZO) films grown by atomic layer deposition**

| Sample | Switching Voltage (V) | Film Thickness (nm) | Preferred Orientation | Reference |
| --- | --- | --- | --- | --- |
| HZO | Does not switch | 17 | UP | ^[1]^ |
| HZO | 9 | 12 | DOWN | ^[2]^ |
| HZO | 10 | 10 | – | ^[3]^ |
| HZO | 9 | 4 – 55 | DOWN | ^[4]^ |
| HZO | 3.5 |  | DOWN | ^[5]^ |
| HZO | 10 |  | UP | ^[6]^ |
| HZO | 3 | 10 | DOWN | ^[7]^ |
| HZO | 10 | 15 | DOWN | ^[8]^ |
| HZO | 7 | 7 | – | ^[9]^ |
| HZO | 8 | 16 | DOWN | ^[10]^ |
| HZO | 20 | 8 | – | ^[11]^ |
| HZO | 5 | 1.5 | DOWN | ^[12]^ |
| HZO | 10 | 10 | – | ^[13]^ |
| HZO | 13 | 15 | UP | ^[14]^ |
| HZO | 9 | 10 | UP | ^[15]^ |
| HZO | 4.8 | 4.5 | – | ^[16]^ |
| HZO | 9 | 20 | – | ^[17]^ |
| HZO | 3 | – | – | ^[18]^ |
| HZO | 2 | 20 | – | This work |

**Supplementary Text 1: Sample cleaning**

The sample cleaning procedure involved four steps: (1) sonicating in isopropanol for 10 minutes, (2) sonicating in acetone for 10 minutes, (3) sonicating in deionized (DI) water for 10 minutes, and (4) drying the samples by blowing with nitrogen gas until completely dry.

**Supplementary Text 2: PFM amplitude variation with humidity and set point**

To compare the piezoelectric behavior of HZO with a conventional ferroelectric material like BFO under varying humidity conditions, we employed cantilever tuning method. This method, which measures the amplitude response at contact resonance through frequency sweeps, provides an effective assessment of the piezoelectric response. Therefore, we conducted frequency sweep measurements on BFO and HZO films in ambient conditions (RH ~ 45%) and a controlled low-humidity environment (RH ~ 1%) to compare the amplitude at the contact resonance frequency. These frequency sweeps were conducted in the range of 200 – 600 kHz, with an applied AC voltage of 6 V and a contact mode set point of 1 V. BFO exhibited higher amplitude under ambient conditions than the low-humidity N_2_ environment. In such conventional ferroelectrics, water and associated ionic species screen depolarization fields, thus stabilizing polarization and enhancing PFM response. Interestingly, the HZO films displayed the opposite trend, with higher amplitude in the N_2_ environment than under ambient conditions as shown in Supplementary Figure 11. The frequency sweeps in low and high humidity conditions were cross-checked at different set points (0.5 V, 1 V, 1.5 V, 2 V), revealing a similar trend for both samples (Supplementary Figure 12). The higher amplitude indicates a stronger piezoelectric response in the controlled N_2_ environment compared to ambient conditions.

**Supplementary Text 3: PFM amplitude variation with humidity and set point**

The strain response of a dielectric material is expressed as^[19]^:

$\varepsilon=Q$ ${c^{2}E}_{a}ⅇ^{ⅈ2\omega t}$ + 2 $Qc{E_{a}(cE}_{0}+P_{s}$) $ⅇ^{ⅈ\omega t}$ (1)

where $c$ is the susceptibility and *E*_0_ is a DC electric field. Equation 1 shows that in materials with strong spontaneous polarization (*P*_s_), the strain response is mainly driven by the first harmonic, which is directly proportional to the applied AC electric field (*E*_a_). Electrostriction (*Q*) contributes to both the first and the second harmonic. In materials with no spontaneous polarization, the second harmonic dominates the strain response. This difference allows us to distinguish between predominantly piezoelectric and non-piezoelectric strain responses by comparing their first and second harmonics.

**Supplementary Text 4: Asymmetry and related phenomena in HZO**

Usually, polarization asymmetry is attributed to interface bias fields, the distribution of oxygen vacancies, depolarization or screening effects, or intrinsic defects^[4,20,21]^. However, in Hf_0.5_Zr_0.5_O_2_ (HZO), such asymmetry has also been linked to factors like film thickness, trapped charges at the interface, and humidity levels^[2,4]^. In our case, the films consistently exhibited a preferred downward polarization orientation both under ambient (after cleaning) and in controlled environments. Given that the film thickness is in a range that provides for strong ferroelectric response and other contributing factors, such as trapped charges at interface, are considered negligible in our model, we propose that a relatively higher concentration of charged oxygen vacancies could plausibly explain the observed preferred orientation.

**Supplementary Text 5: Methodology of ambient atmosphere treatment and XPS**

To investigate the role of atmospheric gas on chemical surface states of HZO films, we performed XPS analysis on samples treated under different atmospheric conditions by adopting a different methodology. Our approach started by exposing the films to N_2_, O_2_, and CO_2_ atmospheres for several hours prior to XPS characterization. The samples were placed in desiccator jars that were evacuated and subsequently backfilled with the respective gases. They were maintained in these controlled environments for 6 hours. It is noteworthy to mention that after exposure, the samples were briefly exposed to ambient air (approximately 5 minutes) before being mounted onto sample platens and loaded into the XPS system.

**Supplementary Text 6: XPS details**

To further investigate the oxidation states of Hf and Zr on the surface of the films, X-ray Photoelectron Spectroscopy (XPS) was employed for the characterization of HZO thin films subjected to different atmospheric treatments. Supplementary Figure 25(a) display the XPS spectra of Hf 4f, Zr 3d, and O 1s peaks recorded from the surfaces of HZO thin films. The Hf 4f and Zr 3d peaks correspond to the Hf⁴⁺ and Zr⁴⁺ oxidation states^[23-25]^.

Furthermore, the deconvolution of the O 1s XPS spectra reveals three distinct peaks, denoted as O1, O2, and O3. The O1 peak corresponds to lattice oxygen present in HfO_2_ and ZrO_2_. The O2 peak is attributed to weakly absorbed oxygen species, or non-lattice oxygen on the surface, whereas the third peak, O3, is ascribed to hydroxyl groups adsorbed on the film surface^[25-27]^. The O2 and O3 peaks reflect deviations from the ideal M:O stoichiometry, suggesting the formation of oxygen vacancies, related defects or adsorbed species.

The quantitative O:Hf and O:Zr ratios are summarized in Supplementary Figure 25(b). These results indicate that oxidation states and oxygen bonding vary with the atmospheric gas. In O_2_, both O:Hf and O:Zr ratios reach their highest values, and the binding energies for Hf 4f_7/2_ (~17.45 eV) and Zr 3d_5/2_ (~182.95 eV) are elevated, indicating enhanced oxidation. In N_2_, the oxidation is weakest, with the lowest ratios and binding energies, suggesting more oxygen vacancies and sub-stoichiometric conditions, whereas CO_2_ gives intermediate level oxidation and binding energy. These findings reveal the role of processing atmosphere in determining the oxidation states of Hf and Zr, which in turn can influence the film's ferroelectric behavior and defect structure.

In the proposed model, we hypothesize that O_2_ effectively passivates oxygen vacancies, which leads to a faster back-transition process. The XPS data support this claim by showing significantly higher oxidation levels under O_2_. Furthermore, when comparing N_2_ and CO_2_, both linear molecules, the model suggests that the higher stability in CO_2_ may stem from its ability to adopt a bent configuration under surface interactions, thereby providing more effective screening, as supported by previous literature^[28]^. Interestingly, this flexibility is indirectly confirmed by XPS observations, which show that CO_2_ leads to higher oxidation than N_2_. The higher oxidation states of metal ions observed in the CO_2_-treated samples compared to those treated in N_2_ may be attributed to the bending of CO_2_ molecules at oxygen vacancy sites. This suggests that CO_2_ can undergo bending upon interacting with charged surfaces, such as those exhibiting surface polarization, thereby contributing to charge screening and higher stability.

**Supplementary Figure 1: Schematic diagram of the ALA HZO process**

**
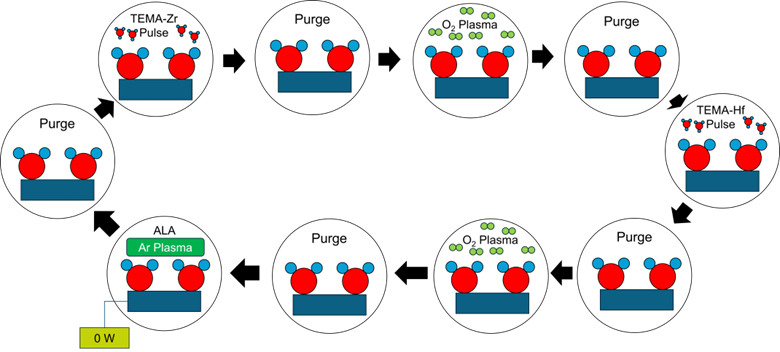
**

**Figure S1.** The blue rectangle represents the substrate. HfO_2_ is deposited using the tetrakis (ethylmethylamido) hafnium (TEMA-Hf) precursor followed by an oxygen plasma. Each HfO_2_ deposition is followed by exposure to argon plasma and a 0 W bias applied to the substrate. ZrO_2_ is deposited using a conventional plasma-enhanced atomic layer deposition process with tetrakis (ethylmethylamido) zirconium (TEMA-Zr).

**Supplementary Figure 2:** **Schematic of HZO samples**


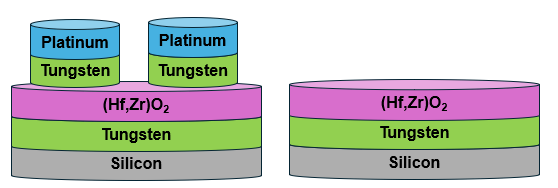


**Figure S2**. The sample on the left was used for grazing-incidence X-ray diffraction and electrical characterization. The sample on the right was used for piezoresponse force microscopy and Fourier transform infrared spectroscopy.

**Supplementary Figure 3:** **X-ray reflectivity spectrum of HZO sample**


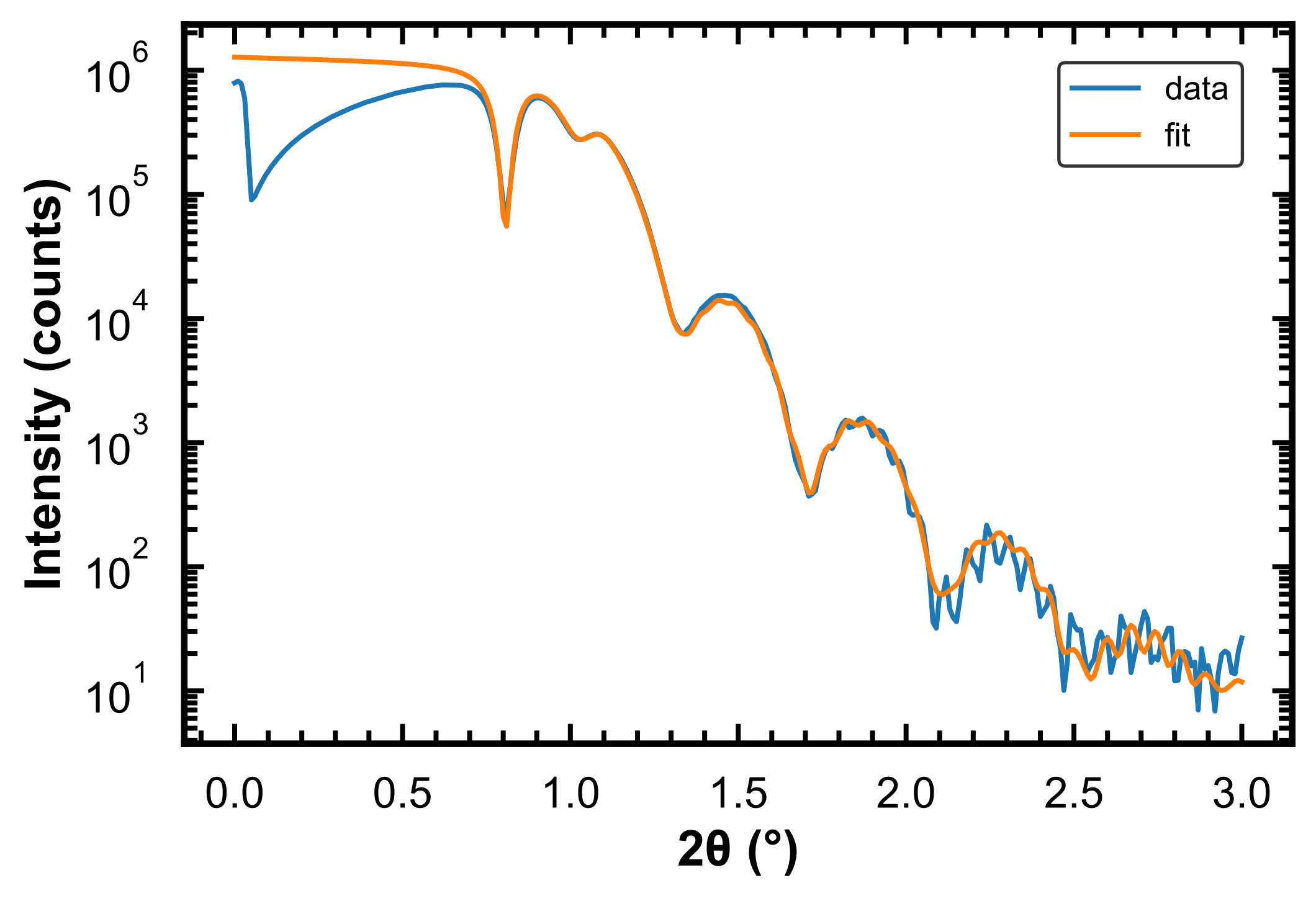


**Figure S3**. X-ray reflectivity pattern (blue line) and theoretical fit (orange line) of HZO stack.

**Supplementary Figure 4:** **XRD pattern of HZO sample with intact top tungsten layer**


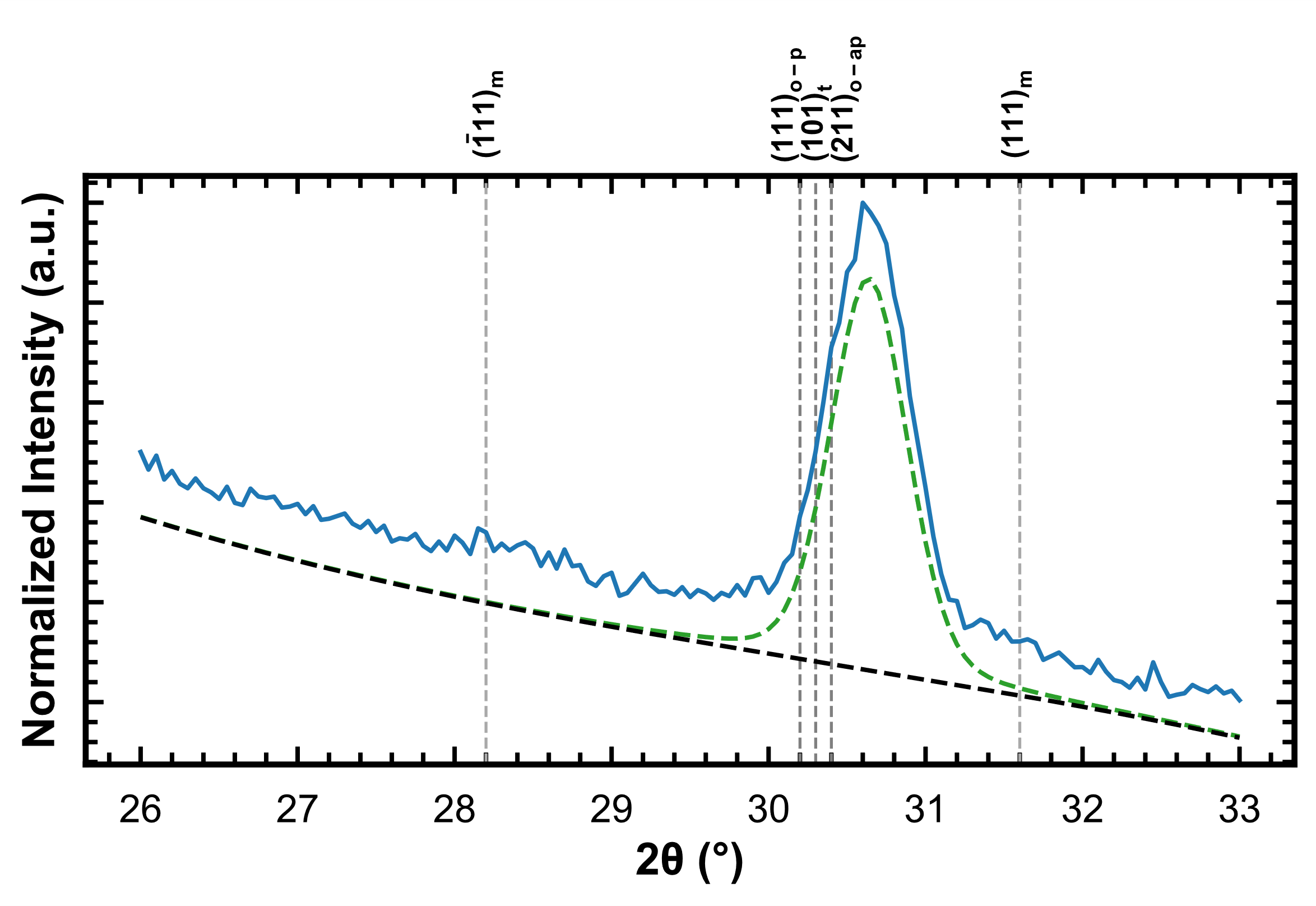


**Figure S4**. XRD pattern of the HZO film with top tungsten layer.

**Supplementary Figure 5:** **FTIR spectrum of HZO sample**


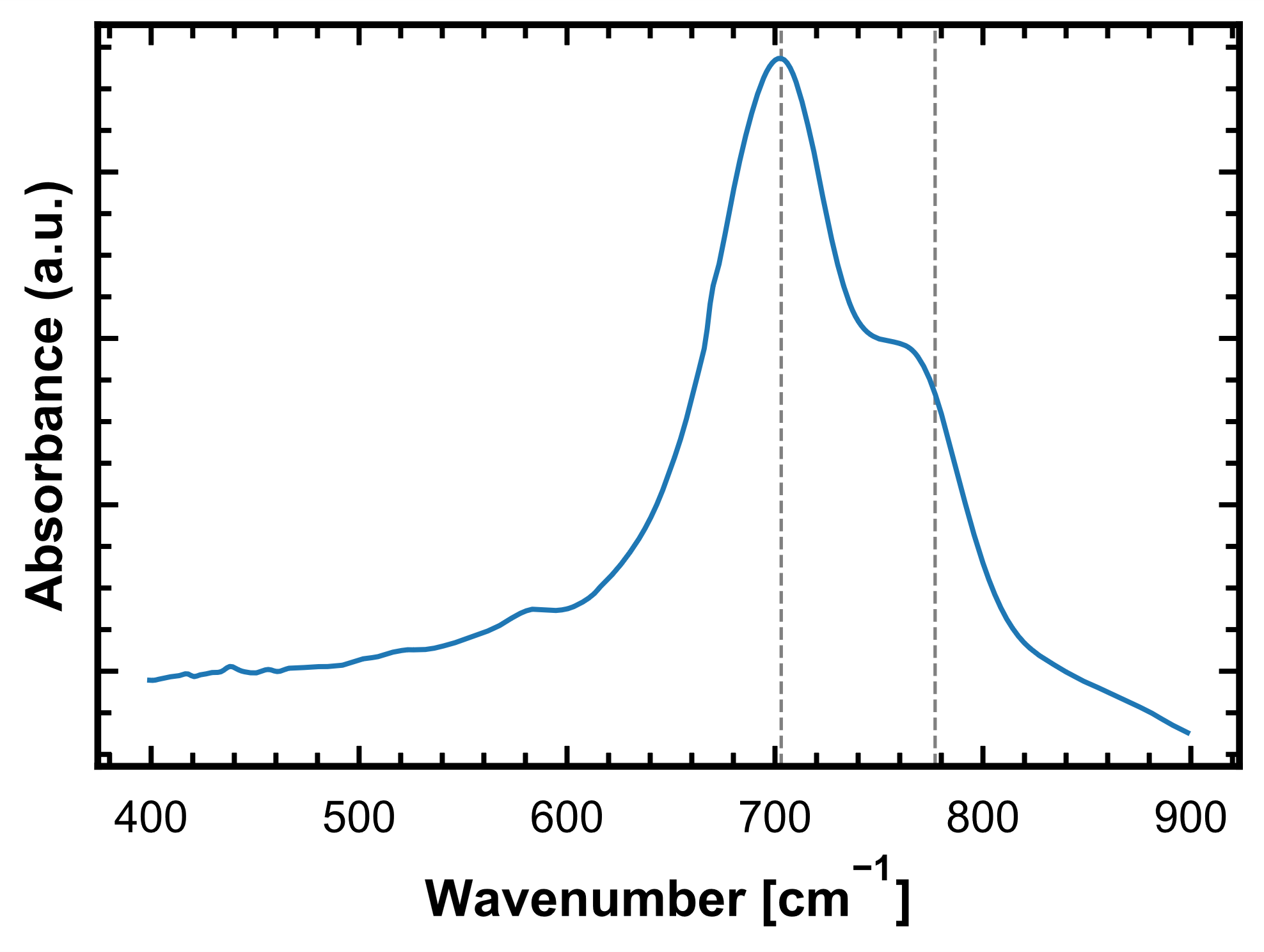


**Figure S5**. FTIR spectrum of annealed sample. The grey dashed lines represent two absorbance modes associated with the polar orthorhombic phase.

**Supplementary Figure 6: HAADF TEM and EDS spectra of HZO sample**

**
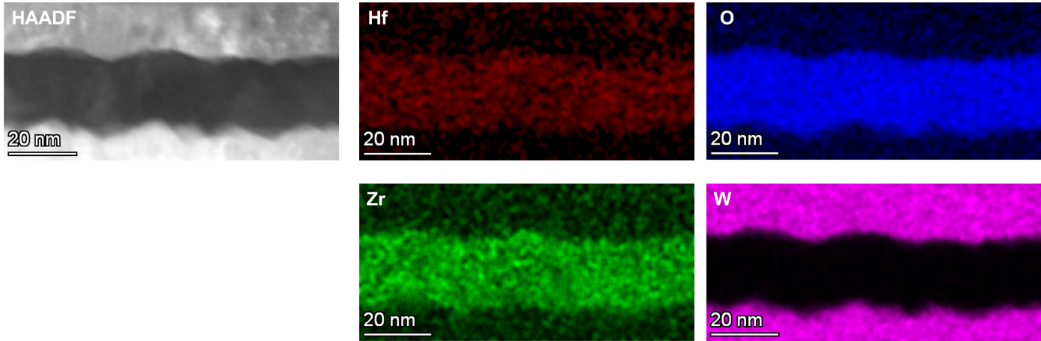
**

**Figure S6.** HAADF TEM and energy dispersive X-ray spectroscopy (EDS) of HZO stack, showing the elemental analysis of the film and electrodes.

**Supplementary Figure 7: XANES spectra of the Hf L^3^ and Hf L_1_ edge**

**
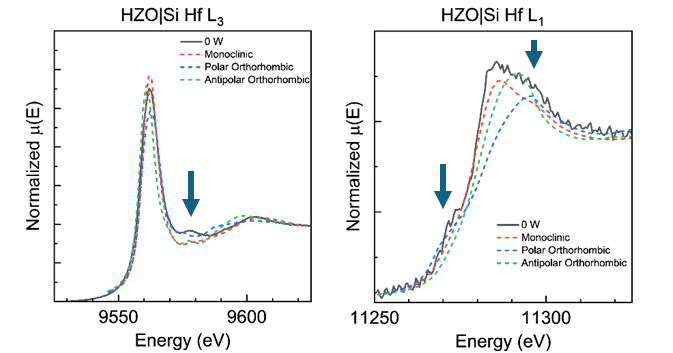
**

**Figure S7.** The XANES region of a given crystal structure serves as a fingerprint for its electronic environment. By comparing FEFF calculations to the experimentally measured spectra, suggestions of the crystal structure can be inferred. The left graph shows the Hf L_3_ edge, which corresponds to the 2*p* → 5*d* transitions. The arrow denotes a feature that could be attributed to a Hf-O-Hf multiple scattering process, which can provide hints at coordination^[22]^. The right graph presents the Hf L_1_ absorption edge, which corresponds to 2*s* → 5*d* transitions. The Hf L_1_ white line exhibits two distinct features that, when compared with FEFF simulations, suggest that the polar orthorhombic phase dominates the film. This conclusion is based on the presence and intensity of the pre-edge peak (first arrow) and the secondary peak on the white line (second arrow).

**Supplementary Figure 8: PUND measurements on HZO capacitors**


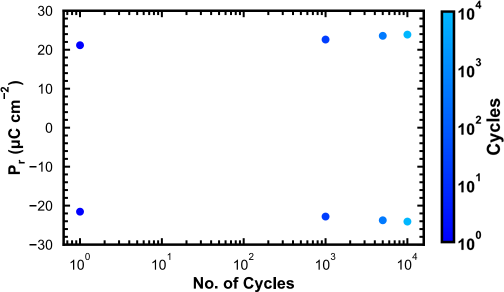


**Figure S8.** Remanent polarization determined from positive up negative down (PUND) measurements taken at each cycling interval versus electric field.

**Supplementary Figure 9: Comparison of PFM response of HZO with a conventional (BiFeO_3_) and non-ferroelectric material (Al_2_O_3_)**

**
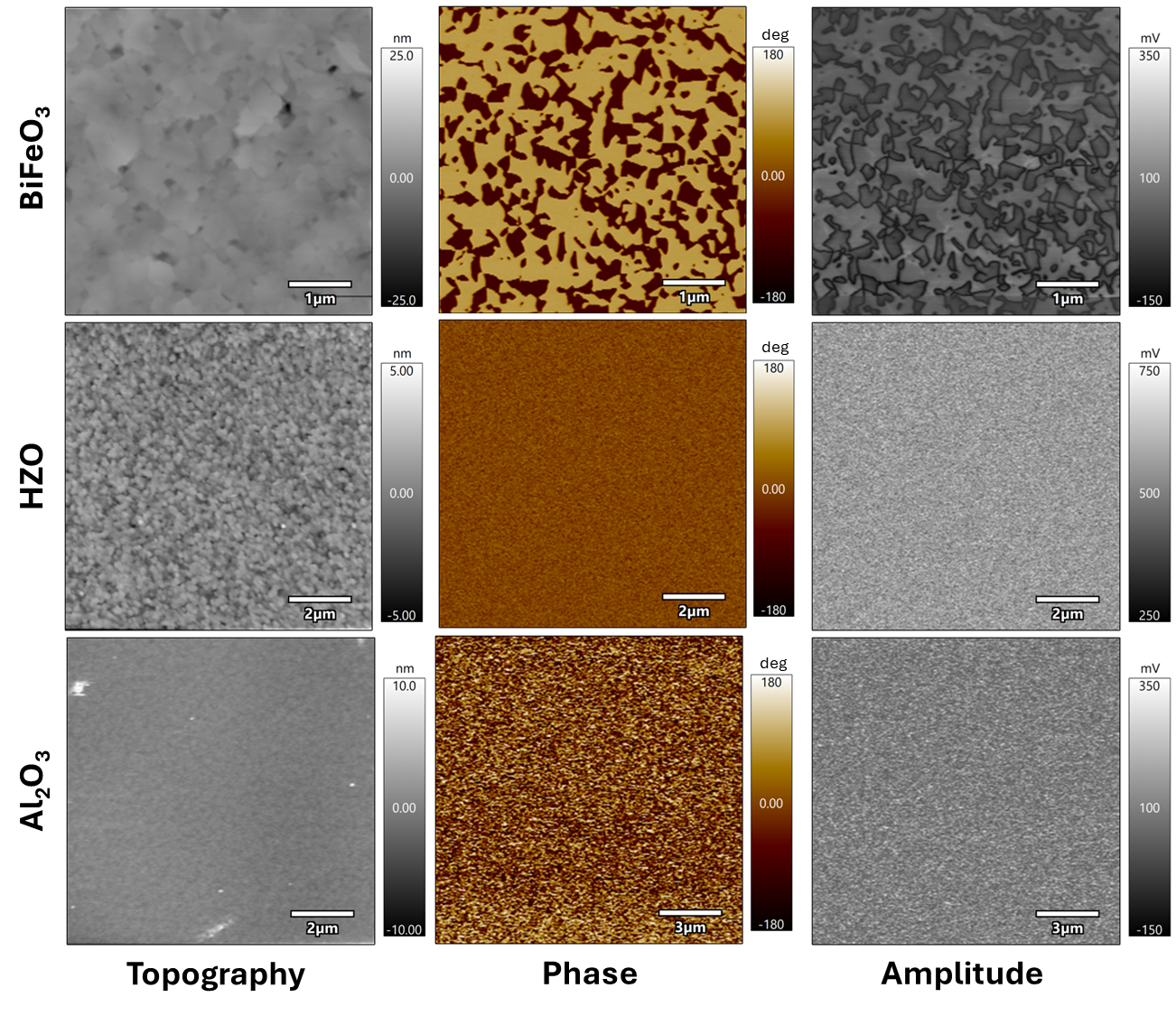
**

**Figure S9.** Topography (left) and PFM phase (middle) and amplitude (right) images of BiFeO_3_ (BFO; 100 nm), Hf_0.5_Zr_0.5_O_2_ (HZO; 20 nm), and Al_2_O_3_ (20 nm) recorded under ambient conditions.

**Supplementary Figure 10: Comparison of domain switching in HZO with a conventional (BiFeO_3_) and non-ferroelectric material (Al_2_O_3_) in ambient conditions**

**
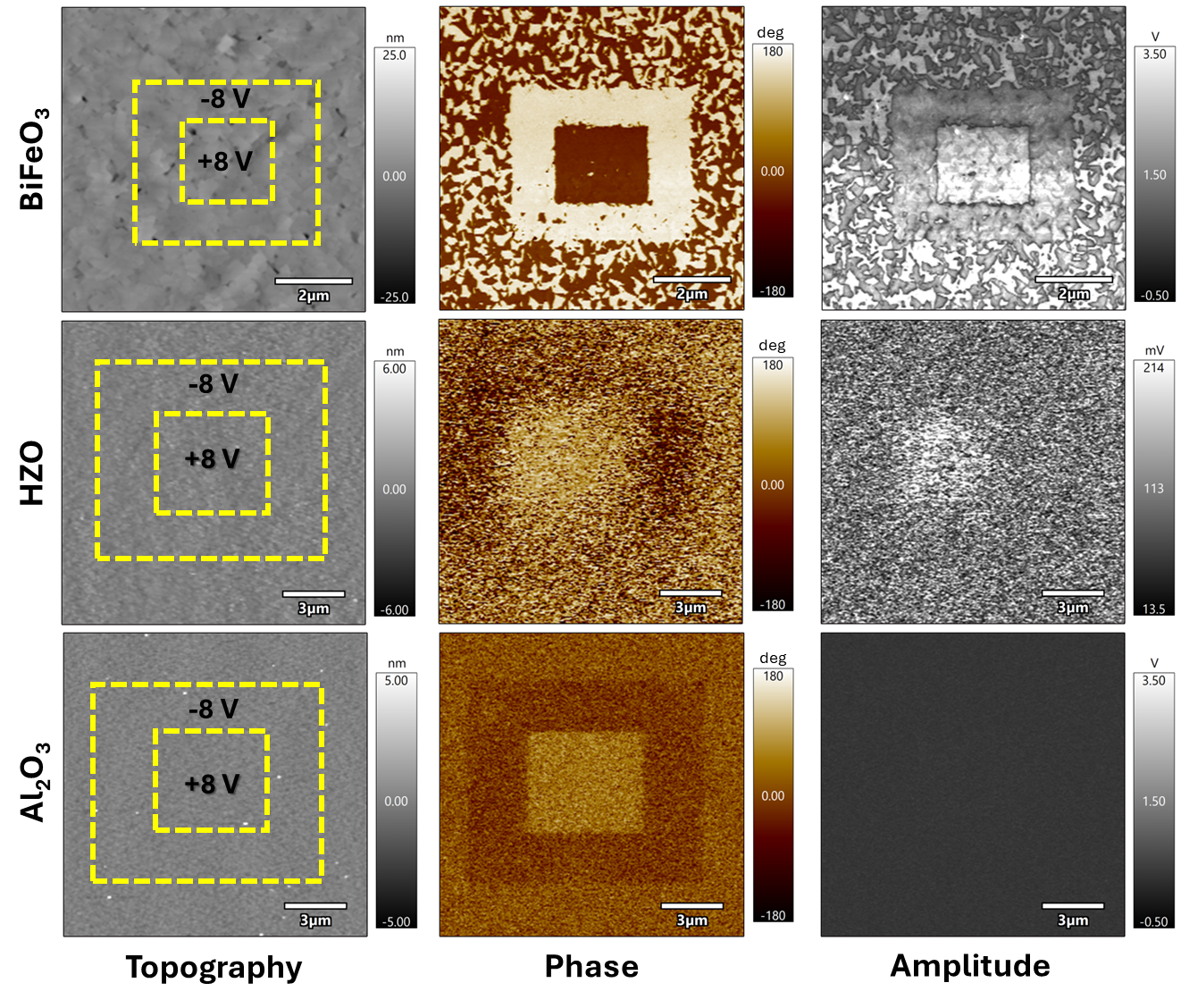
**

**Figure S10.** Domain switching (topography-left, phase-middle, amplitude-right) of BFO, HZO and Al_2_O_3_, at an applied bias of ± 8 V under ambient conditions (RH = 45%).

**Supplementary Figure 11: Comparison of amplitude in low and high humidity conditions for HZO and BiFeO**_3_

**
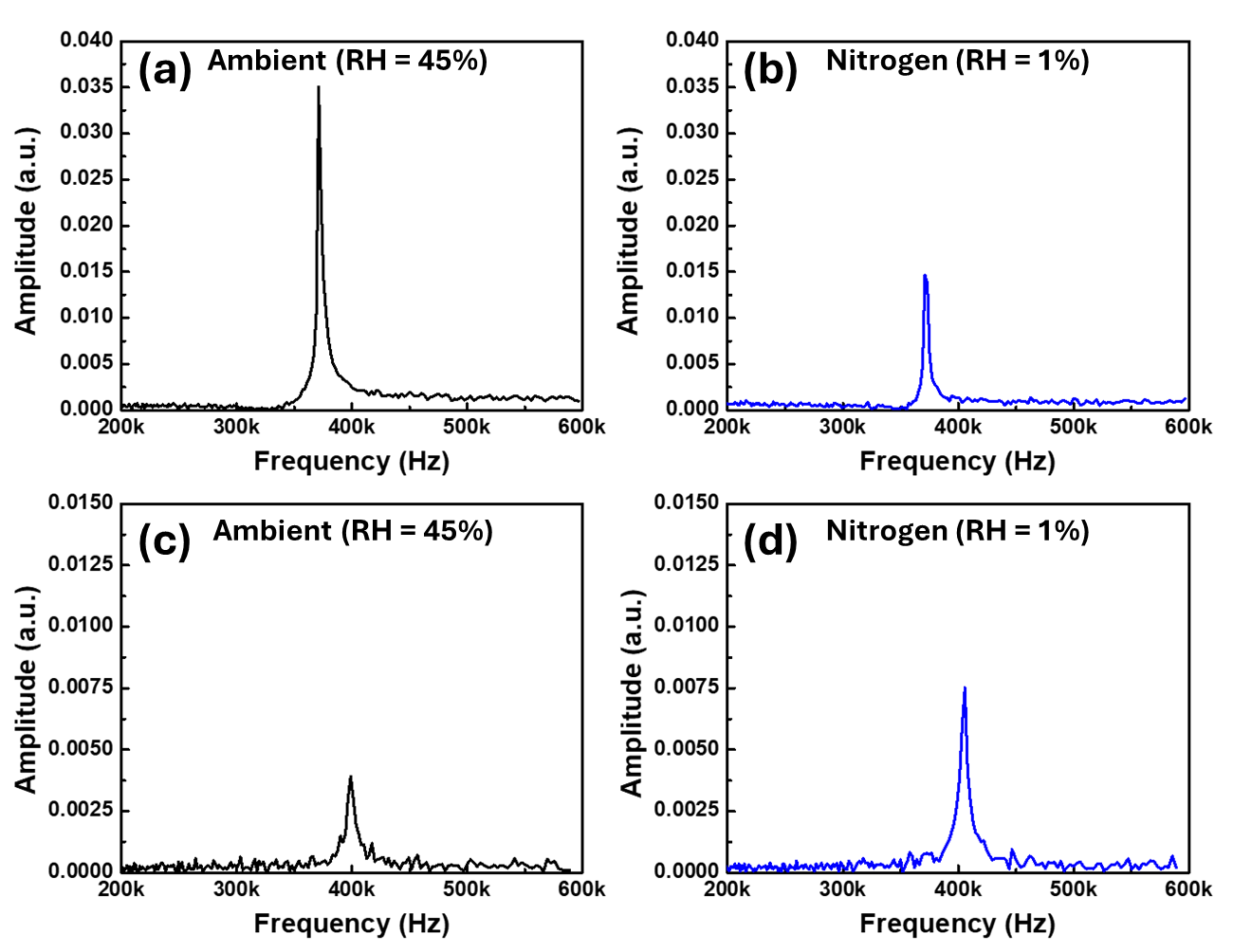
**

**Figure S11.** Frequency sweep of PFM amplitude recorded on BFO under (a) ambient conditions and in (b) N_2_; frequency sweep of HZO in (c) ambient conditions and (d) N_2_ environment.

**Supplementary Figure 12: Variation of PFM amplitude response of HZO with set point**

**
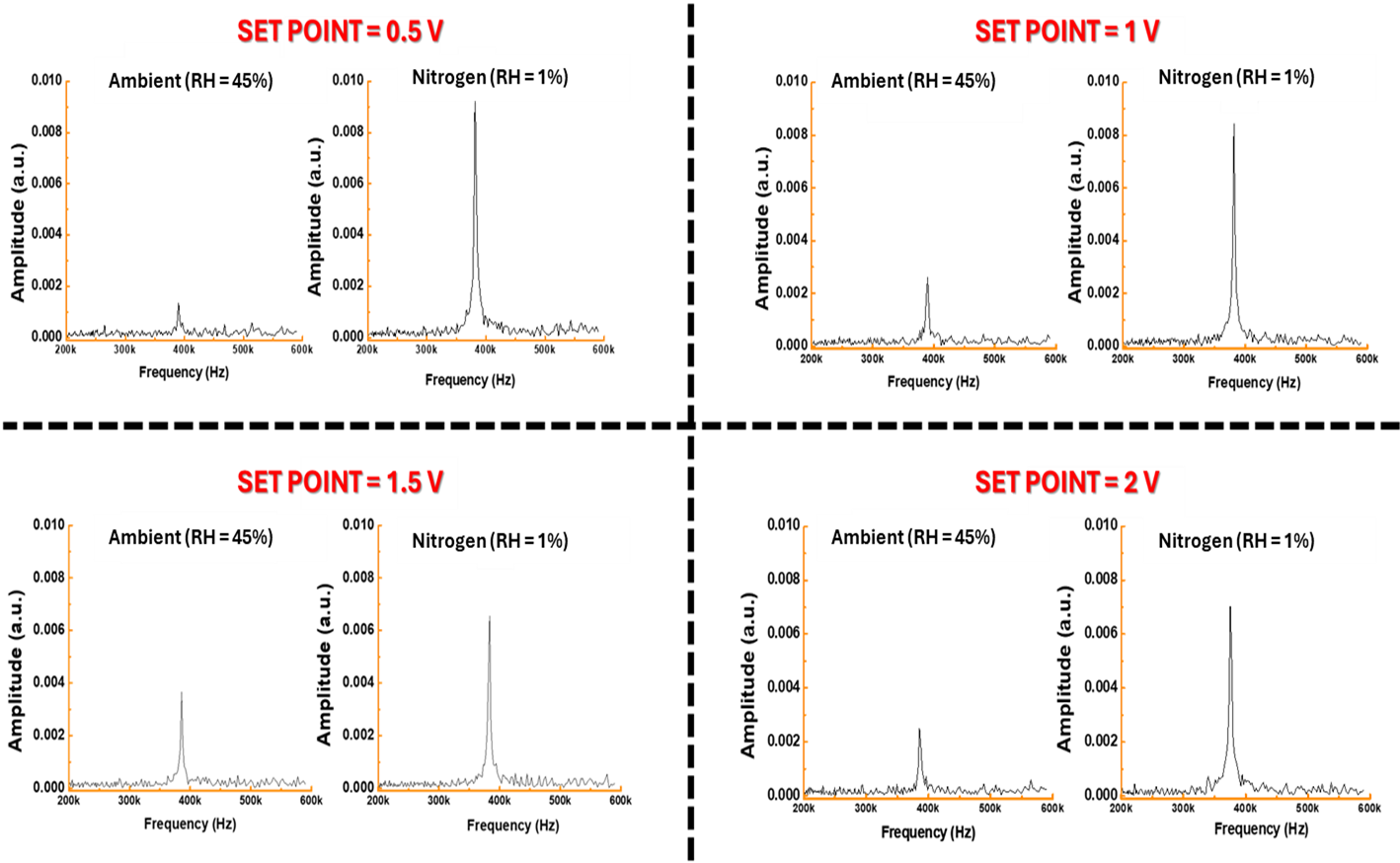
**

**Figure S12.** Frequency sweeps of PFM amplitude recorded on HZO at different loading forces in ambient and nitrogen atmospheres. The forces corresponding to the set point of 0.5 V, 1 V, 1.5 V and 2 V were measured to be 136 ± 5 nN, 272 ± 11 nN, 409 ± 16 nN, and 545 ± 22 nN, respectively.

**Supplementary Figure 13: PFM of BiFeO_3_ in nitrogen environment (RH = 1%)**

**
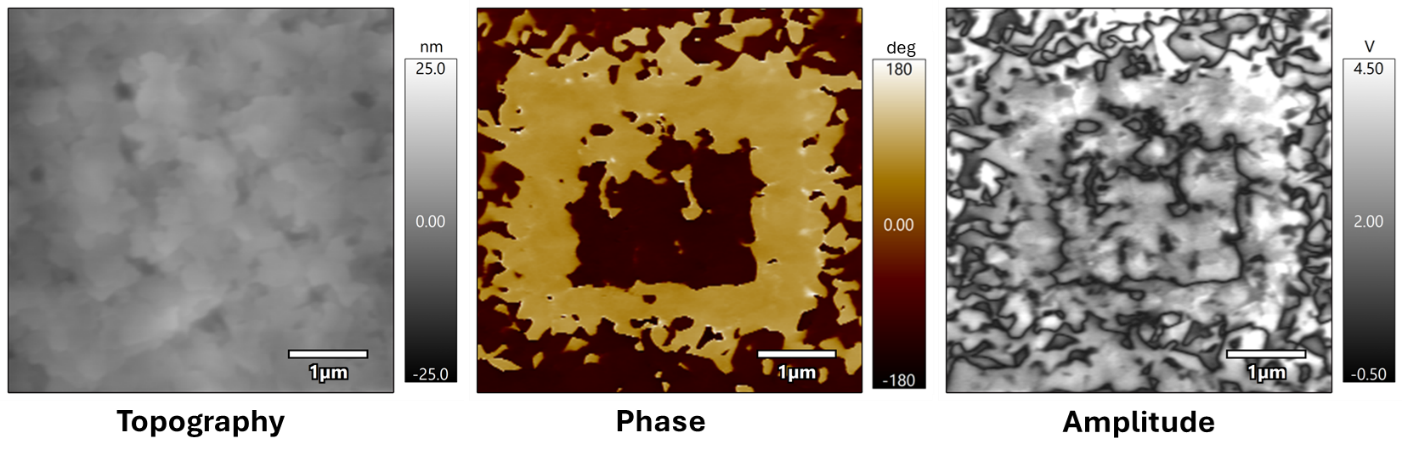
**

**Figure S13.** Topography (left) and PFM phase (middle) and amplitude (right) images of BFO after box-in-box switching with ± 10 V (which is comparatively higher than in ambient conditions ±8 V) in nitrogen at a RH of 1%.

**Supplementary Figure 14: PFM phase and amplitude images of domains switched at different voltages**


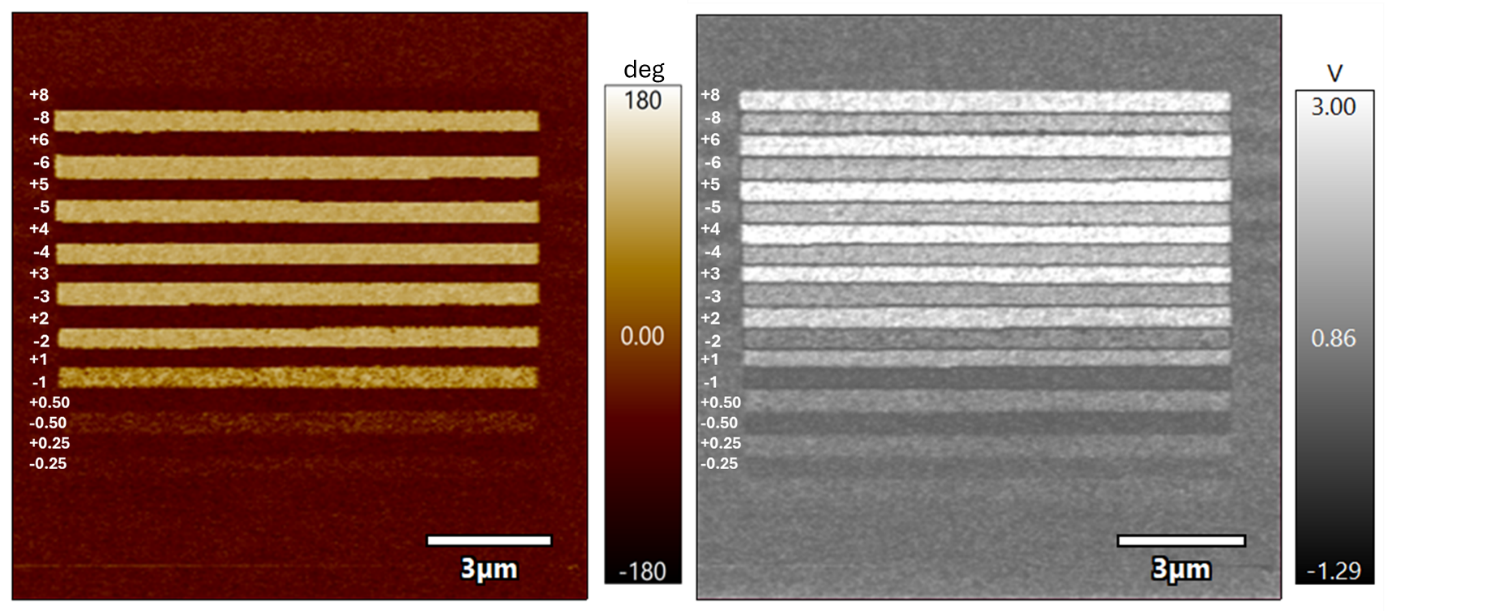


**Figure S14.** PFM phase and amplitude images of domains at different applied voltages (± 0.25 V to ± 8 V).

**Supplementary Figure 15: Domain writing and reading at opposite interfaces**


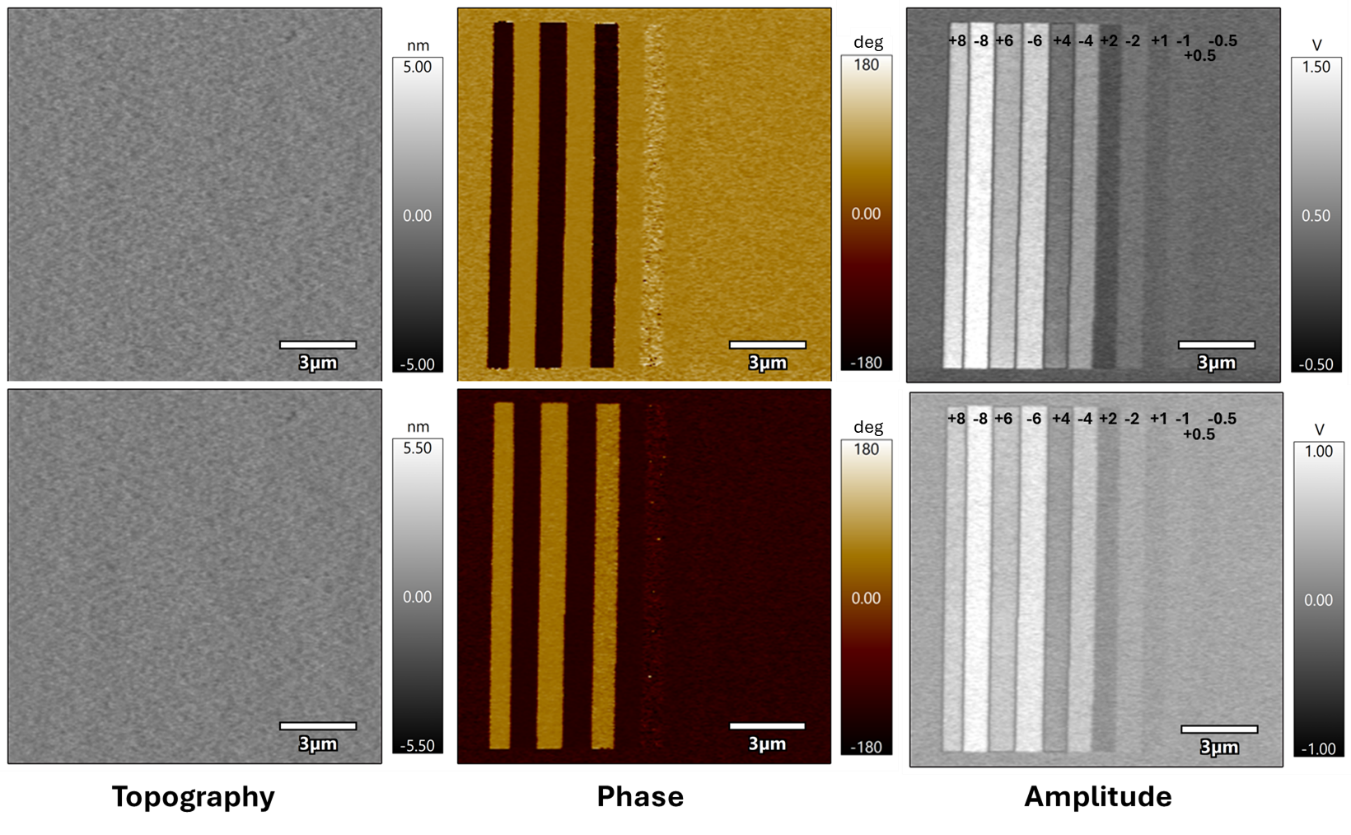


**Figure S15.** (a) Topography and PFM images showing the response after domain writing using the bottom electrode (bottom surface) and measuring the PFM response through the same electrode. (b) Topography and PFM images showing the response after domain writing using the bottom electrode (bottom surface) and measuring the PFM response via the tip (top surface).

**Supplementary Figure 16: First and second harmonic AC voltage sweeps**


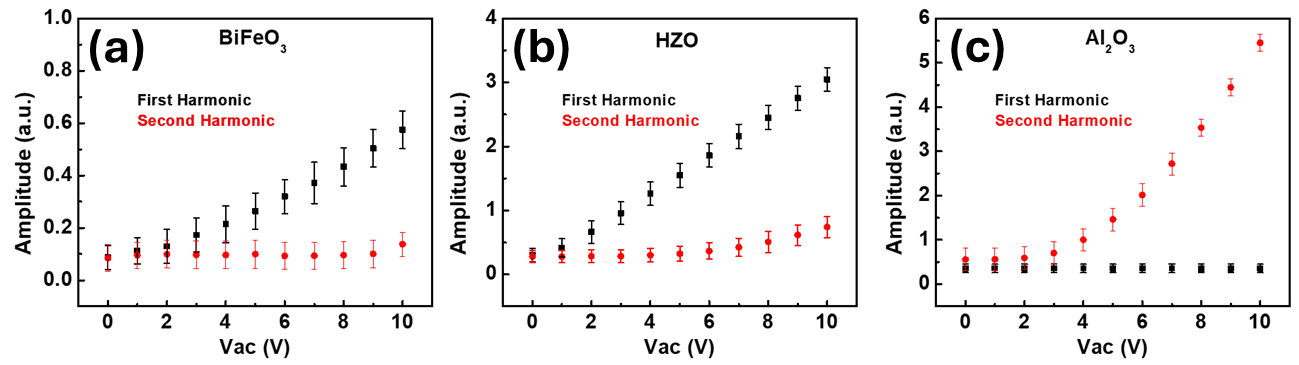


**Figure S16.** First and second harmonic AC voltage sweeps for (a) BiFeO_3_, (b) HZO, and (c) Al_2_O_3_.

**Supplementary Figure 17: Piezoelectric coefficient (d_33_) measurement**

**Figure S17.** The piezoelectric coefficient (d_33_) was calculated from the slope of amplitude vs Vac plots using the relation ε = d_33_V_ac_. A representative plot is shown. The average value of 10 such measurements across three locations was measured to be 1.8 ± 0.2 pm/V. It is important to note that the measurements were taken near the contact resonance. To account for resonance amplification, we divided the results by the quality factor of the amplitude peak at resonance.

**Supplementary Figure 18: Domain stability in CO_2_ environment at RH = 3% with fewer scans**

**
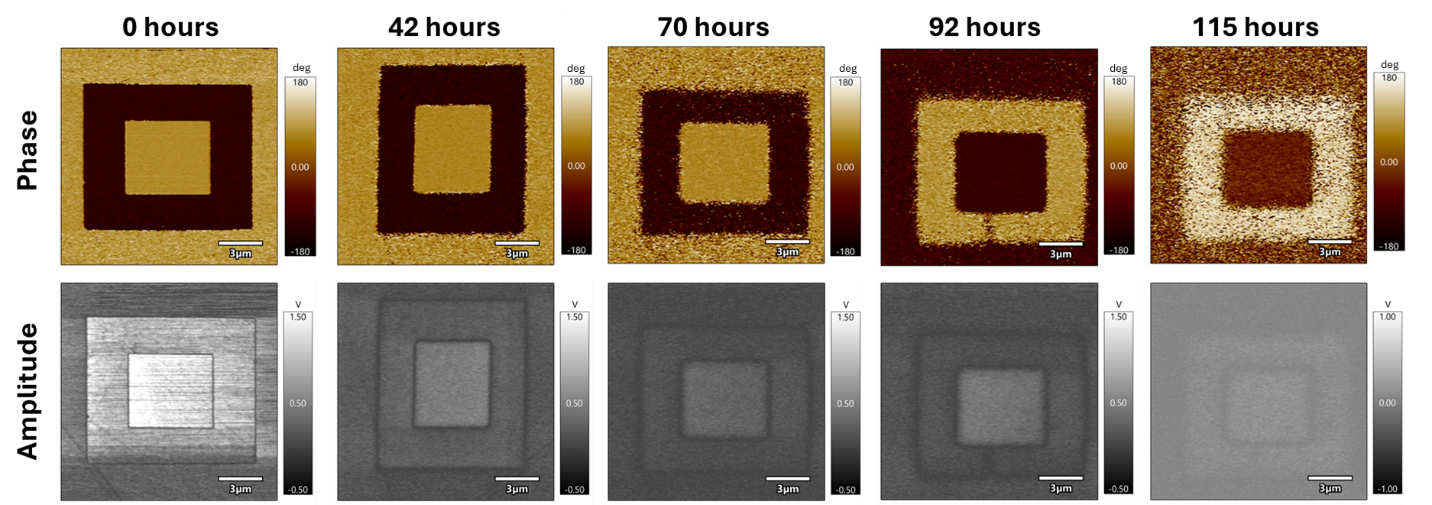
**

**Figure S18.** Domain stability of HZO in CO_2_ with fewer scans than Fig. 4.

**Supplementary Figure 19: Stability variation with the number of scans**


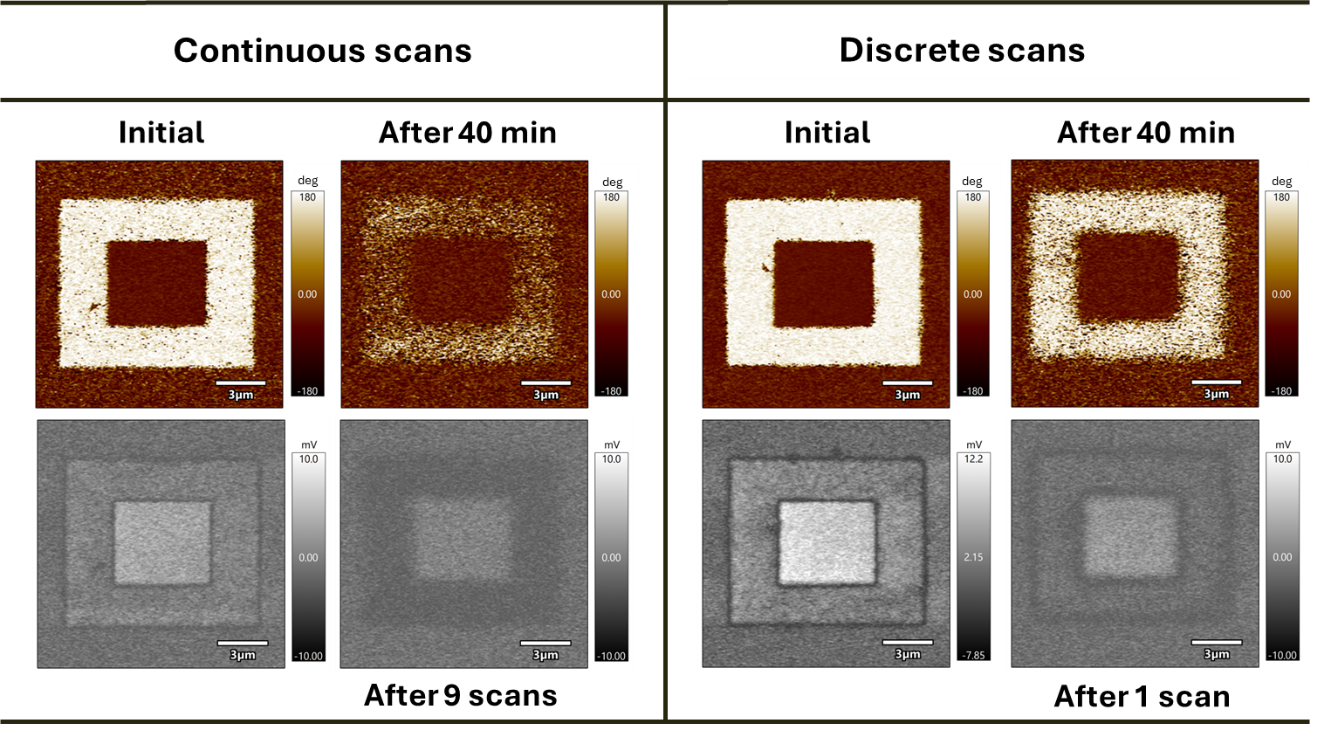


**Figure S19.** Comparison of domain relaxation with continuous (left) vs discrete scans (right) in nitrogen environment.

**Supplementary Figure 20: Amplitude variation with time**

**
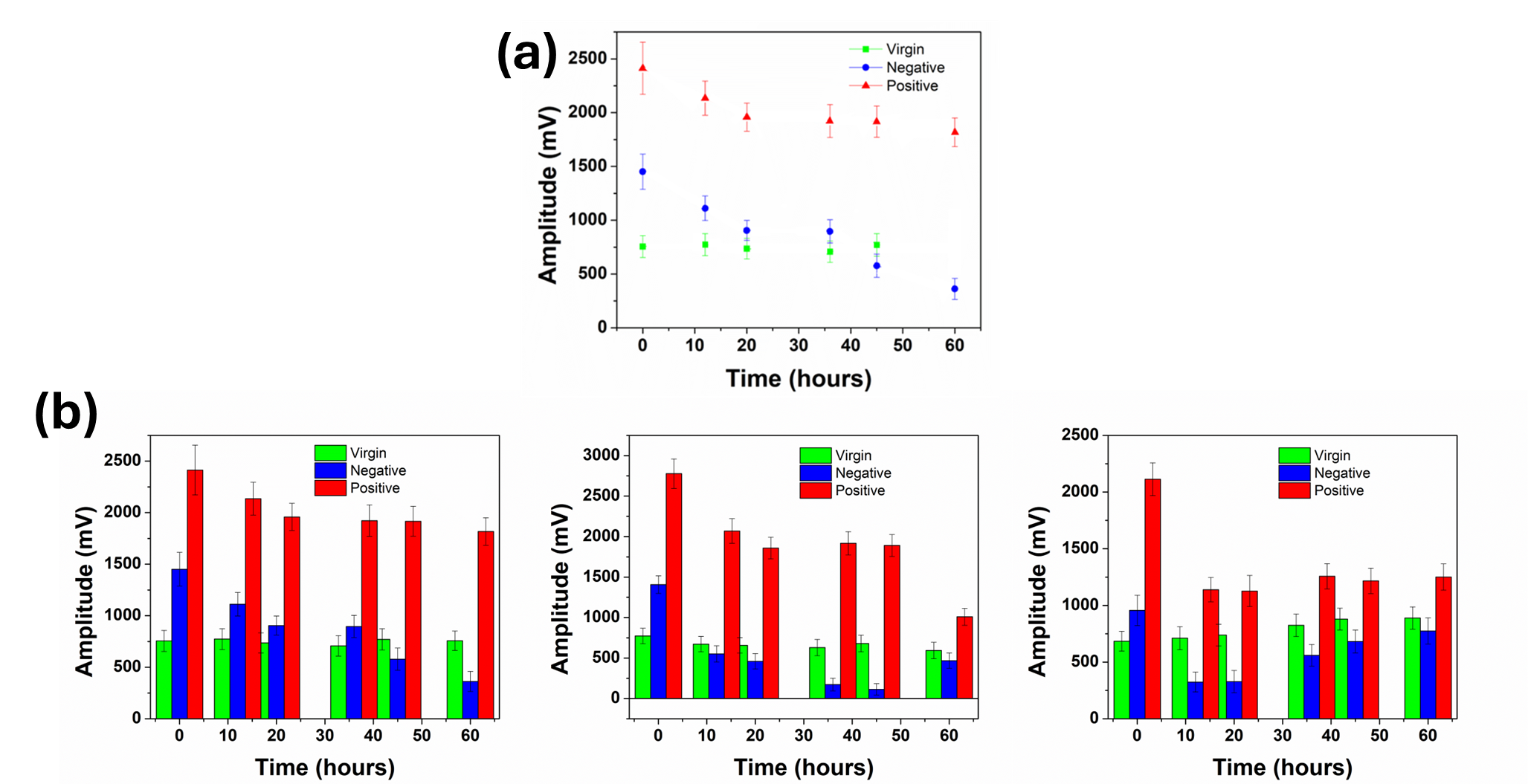
**

**Figure S20.** (a) Amplitude variation over time in CO_2_ for ± 8 V. (b) Comparison of virgin, negative and positive bias applied regions poled at (left) ± 8 V, (middle) ± 6 V, and (right) ± 4 V.

**Supplementary Figure 21: Comparison of minimum poling voltage and stability in dry and wet oxygen**


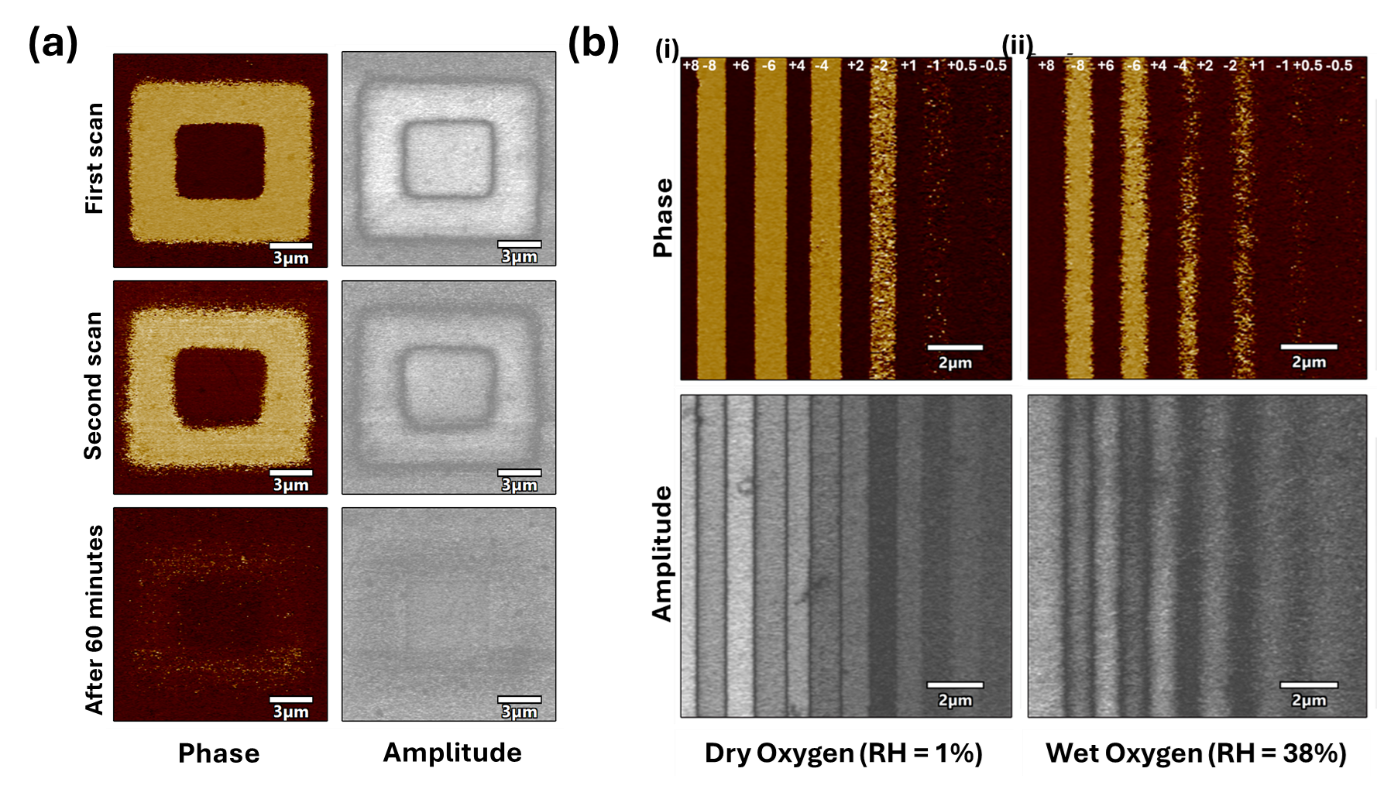


**Figure S21.** (a) Stability comparison when switched at ±8 V and (b) switching voltage bias comparison in (i) dry and (ii) wet oxygen. The wet oxygen environment was prepared by passing oxygen through deionized water using a custom-built setup, followed by its introduction into the humidity chamber.

**Supplementary Figure 22: Exposure to ambient atmosphere after switching at low humidity**


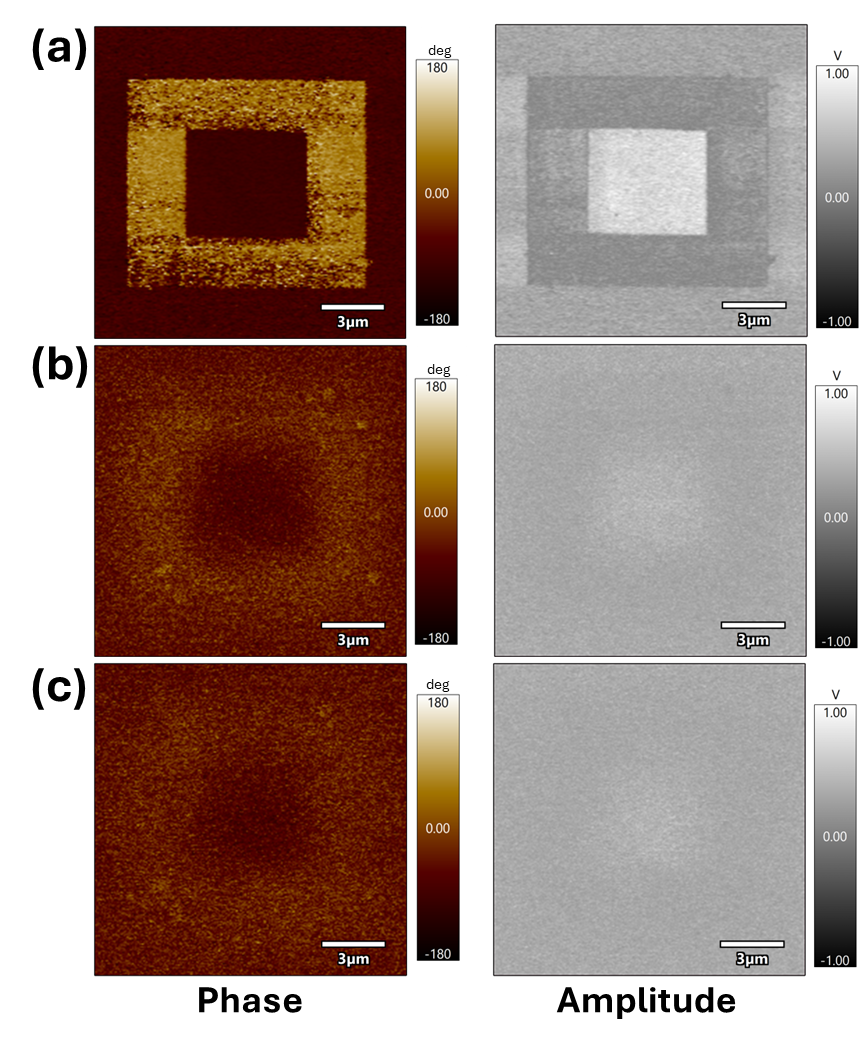


**Figure S22.** PFM phase (left) and amplitude (right) images after stopping the gas supply: (a) first scan, (b) second scan, and (c) third scan.

**Supplementary Figure 23: Surface charge dissipation in ambient conditions**


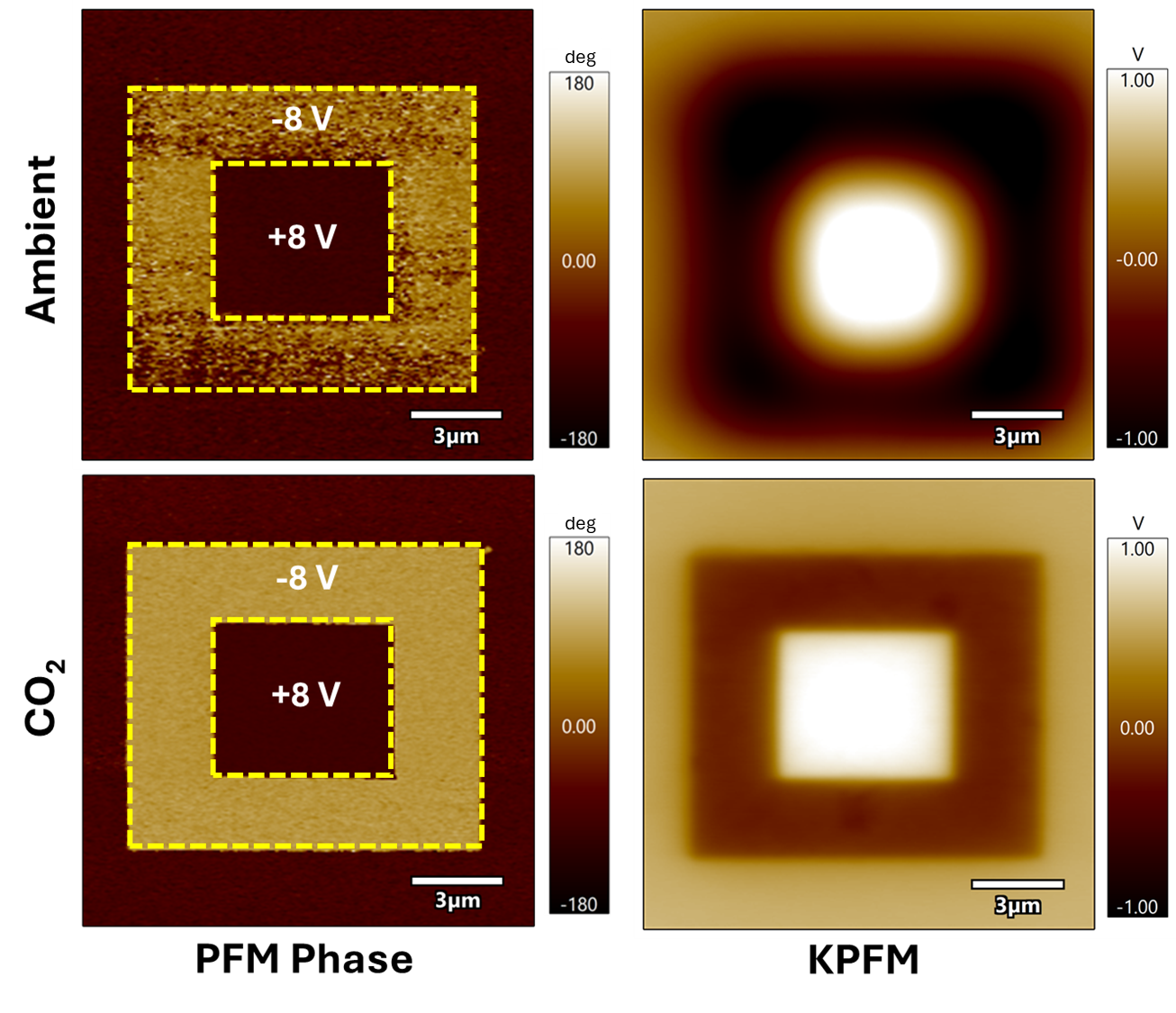


**Figure S23.** PFM phase (left) and KPFM images (right) in ambient (top) and CO_2_ environments (bottom).

**Supplementary Figure 24: Force curves in ambient and CO_2_ environment conditions**

**
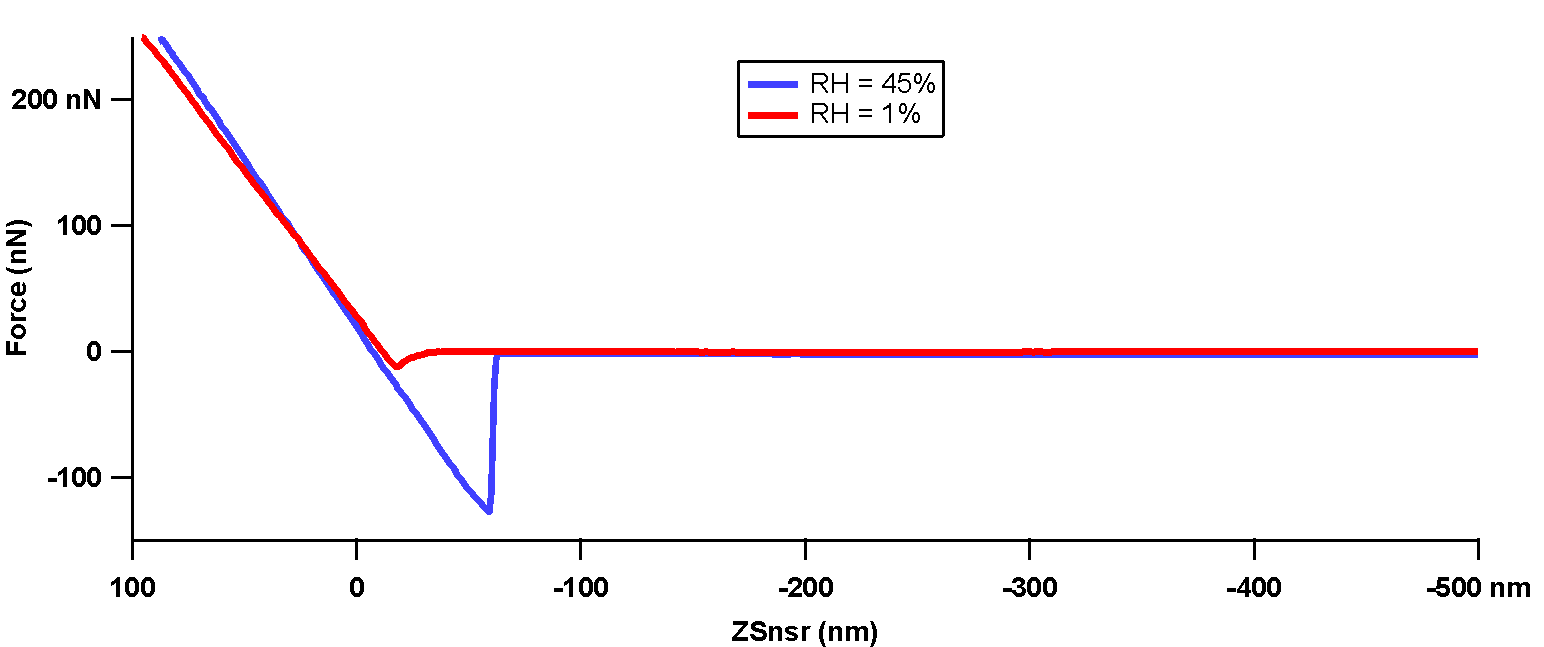
**

**Figure S24.** Retract force-distance curves in ambient (RH = 45%, blue) and CO_2_ (RH = 1%, red) environments. Each curve is an average of 20 force curves.

**Supplementary Figure 25: XPS results**


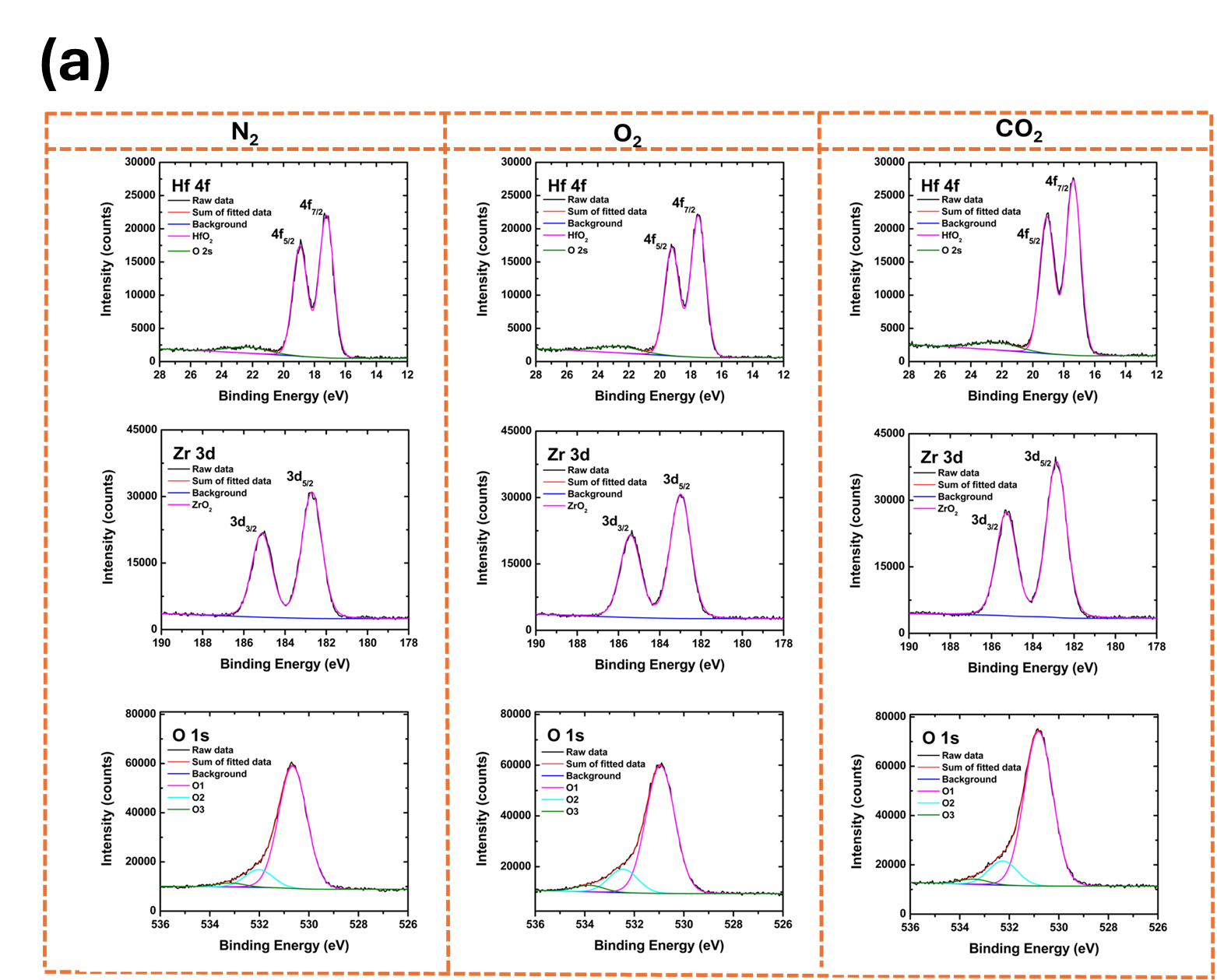


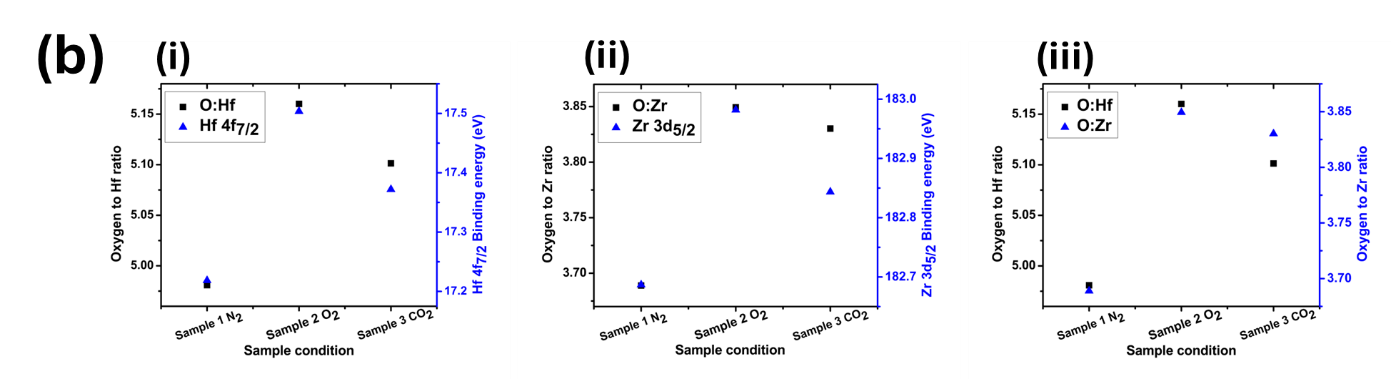


**Figure S25.** (a) XPS spectra of Hf, Zr, and O in HZO films after atmospheric treatment in N₂, O₂, and CO₂ environments. (b) Summary of surface composition and binding energy extracted from XPS analysis showing (i) ratio of O:Hf, (ii) ratio of O:Zr, and (iii) comparison of O:Hf and O:Zr ratios after exposure to different gas atmospheres.

**Supplementary Figure 26: Injected charge variation with applied bias voltage**

**
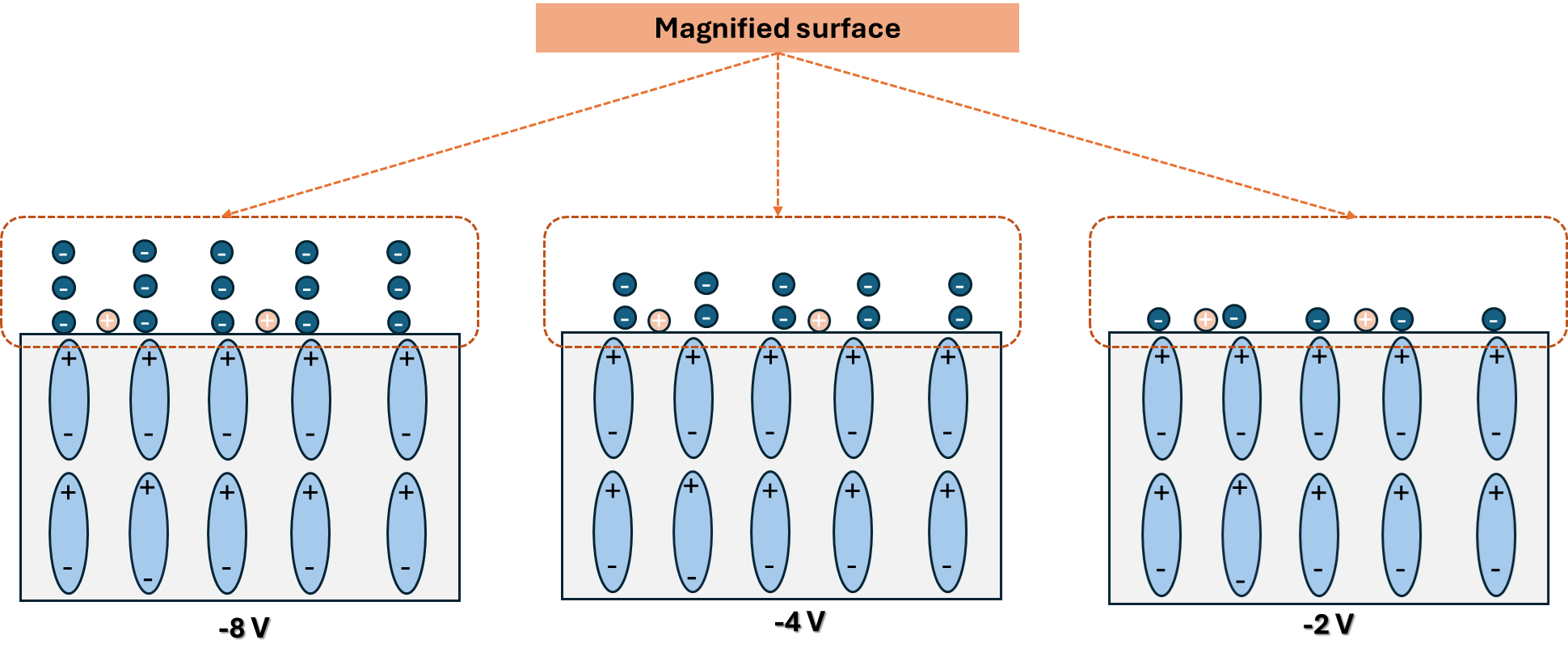
**

**Figure S26.** Schematic of injected charge concentration with applied bias voltage.

**References**

[1] K. P. Kelley, A. N. Morozovska, E. A. Eliseev, Y. Liu, S. S. Fields, S. T. Jaszewski, T. Mimura, S. Calderon, E. C. Dickey, J. F. Ihlefeld, S. V. Kalinin, *Nat Mater* 2023, *22*, 1144. DOI 10.1038/s41563-023-01619-9.

[2] L. Q. Wei, Z. Guan, W. Y. Tong, W. C. Fan, A. Mattursun, B. Bin Chen, P. H. Xiang, G. Han, C. G. Duan, N. Zhong, *Advanced Science* 2024, DOI 10.1002/advs.202410354.

[3] A. Frechilla, M. Napari, N. Strkalj, E. Barriuso, K. Niang, M. Hellenbrand, P. Strichovanec, F. M. Simanjuntak, G. Antorrena, A. Flewitt, C. Magén, G. F. de la Fuente, J. L. MacManus-Driscoll, L. A. Angurel, J. Á. Pardo, *Appl Mater Today* 2024, *36*, DOI10.1016/j.apmt.2023.102033.

[4] Y. Zhu, H. Ning, Z. Yu, Q. Pan, C. Zhang, C. Luo, X. Tu, Y. You, P. Wang, X. Wu, Y. Shi, X. Wang, *Adv Electron Mater* 2019, *5*, DOI 10.1002/aelm.201900554.

[5] T. Kim, G. Kim, Y. K. Lee, D. H. Ko, J. Hwang, S. Lee, H. Shin, Y. Jeong, S. O. Jung, S. Jeon, *Adv Funct Mater* 2023, *33*, DOI 10.1002/adfm.202208525.

[6] C. Liu, Q. Yang, B. Zeng, Y. Jiang, S. Zheng, J. Liao, S. Dai, X. Zhong, Y. Zhou, M. Liao, *Adv Funct Mater* 2022, *32*, DOI 10.1002/adfm.202209604.

[7] Z. Xiao, H. Y. Yoong, J. Cao, Z. Zhao, J. Chen, X. Yan, *Advanced Intelligent Systems* 2022, *4*, DOI 10.1002/aisy.202100244.

[8] W. Y. Liu, J. J. Liao, J. Jiang, Y. C. Zhou, Q. Chen, S. T. Mo, Q. Yang, Q. X. Peng, L. M. Jiang, *J Mater Chem C Mater* 2020, *8*, 3878, DOI 10.1039/C9TC05157K.

[9] S. P. Chiniwar, Y. C. Hsieh, C. H. Shih, C. Y. Teng, J. L. Yang, C. Hu, B. H. Lin, M. T. Tang, Y. C. Tseng, *ACS Appl Electron Mater* 2024, *6*, 1078, DOI 10.1021/acsaelm.3c01502.

[10] J. Liao, T. Yang, C. Ju, Q. Yang, M. Liao, B. Zeng, Y. Zhou, *J Phys D Appl Phys* 2023, *56*, DOI 10.1088/1361-6463/acdaa4.

[11] Z. Zou, G. Tian, D. Wang, Y. Zhang, J. Wang, Y. Li, R. Tao, Z. Fan, D. Chen, M. Zeng, X. Gao, J. Y. Dai, X. Lu, J. M. Liu, *Nanotechnology* 2021, *32*, DOI 10.1088/1361-6528/abfc70.

[12] Z. Gao, W. Zhang, Q. Zhong, Y. Zheng, S. Lv, Q. Wu, Y. Song, S. Zhao, Y. Zheng, T. Xin, Y. Wang, W. Wei, X. Ren, J. Yang, C. Ge, J. Tao, Y. Cheng, H. Lyu, *Device* 2023, *1*, DOI 10.1016/j.device.2023.100004.

[13] B. Liu, Y. Zhang, L. Zhang, Q. Yuan, W. Zhang, Y. Li, *J Alloys Compd* 2022, *919*, DOI 10.1016/j.jallcom.2022.165872.

[14] C. Ju, B. Zeng, Z. Luo, Z. Yang, P. Hao, L. Liao, Q. Yang, Q. Peng, S. Zheng, Y. Zhou, M. Liao, *Journal of Materiomics* 2024, *10*, 277, DOI: 10.1016/j.jmat.2023.05.013

[15] Y. Cao, W. Zhang, Y. Li, *Nanoscale* 2022, *15*, 1392, DOI: 10.1039/D2NR05678J

[16] P. Chaudhary, P. Buragohain, M. Kozodaev, S. Zarubin, V. Mikheev, A. Chouprik, A. Lipatov, A. Sinitskii, A. Zenkevich, A. Gruverman, *Appl Phys Lett* 2021, *118*, DOI 10.1063/5.0035306.

[17] Y. Zhang, Z. Fan, D. Wang, J. Wang, Z. Zou, Y. Li, Q. Li, R. Tao, D. Chen, M. Zeng, X. Gao, J. Dai, G. Zhou, X. Lu, J. M. Liu, *ACS Appl Mater Interfaces* 2020, *12*, 40510, DOI 10.1021/acsami.0c10964.

[18] T. Kim, J. Park, B. H. Cheong, S. Jeon, *Appl Phys Lett* 2018, *112*, DOI 10.1063/1.5003369.

[19] J. Yu, E. N. Esfahani, Q. Zhu, D. Shan, T. Jia, S. Xie, J. Li, *J Appl Phys* 2018, *123*, DOI 10.1063/1.5023407.

[20] H. Lee, T. H. Kim, J. J. Patzner, H. Lu, J. W. Lee, H. Zhou, W. Chang, M. K. Mahanthappa, E. Y. Tsymbal, A. Gruverman, C. B. Eom, *Nano Lett* 2016, *16*, 2400, DOI: 10.1021/acs.nanolett.5b05188.

[21] N. Balke, R. Ramesh, P. Yu, *ACS Appl Mater Interfaces* 2017, *9*, 39736, DOI :10.1021/acsami.7b10747.

[22] L. Botti, S. A. Kondrat, R. Navar, D. Padovan, J. S. Martinez-Espin, S. Meier, C. Hammond, *Angewandte Chemie - International Edition* 2020, *59*, 20017, DOI: 10.1002/anie.202006718.

[23] M. O. Hill, J. S. Kim, M. L. Müller, D. Phuyal, S. Taper, M. Bansal, M. T. Becker, B. Bakhit, T. Maity, B. Monserrat, G. D. Martino, *Adv. Mater.* 2024, *36*, 2408572, DOI:10.1002/adma.202408572

[24] C. An, J. W. Cho, T. Y. Lee, M. S. Song, B. Kang, H. Kim, J. H. Lee, C. Sohn, S. C. Chae, Adv. Mater. Interfaces 2025, 12, 2400742, DOI: 10.1002/admi.202400742

[25] K. Yang, G.‑Y. Kim, J. J. Ryu, D. H. Lee, J. Y. Park, S. H. Kim, G. H. Park, G. T. Yu, G. H. Kim, S. Y. Choi, M. H. Park, Adv. Mater. 2023, 35, 2302585, DOI: 10.1002/adma.202302585.

[26] M. Mayorga‑Garay, O. Cortazar‑Martinez, D. P. Silvas‑Cabrales, J. A. Torres‑Ochoa, F. Corona‑Davila, J. A. Carmona‑Carmona, D. M. Guzmán‑Bucio, A. Herrera‑Gomez, Appl. Surf. Sci. 2024, 678, 161073, DOI: 10.1016/j.apsusc.2024.161073.

[27] T. V. Perevalov, I. P. Prosvirin, E. A. Suprun, F. Mehmood, T. Mikolajick, U. Schroeder, V. A. Gritsenko, *J. Sci. Adv. Mater. Devices* 2021, *6*, 595–600, DOI: 10.1016/j.jsamd.2021.08.001.

[28] Yu, J. Li, Y. Zhang, S. Yang, K. Han, F. Dong, T. Ma, H. Huang, *Angew. Chem. Int. Ed*., 2019, 58 (12), 3880–3884, DOI: 10.1002/anie.201813967.
