# Supplementary material for: Environmental Control of Ferroelectricity in Hafnia Films
Source: Adv Mater. 2025 Aug 7;37(42):e03852. doi: 10.1002/adma.202503852 (PMC12548525; doi:10.1002/adma.202503852)
Supplement: Supplementary file 2 [file ADMA-37-e03852-s001.odt]

- P-E Measurements in Different Environments:

As part of the current work, we performed bulk polarization–electric field (P–E) measurements under different environmental conditions (O2, N2, CO2, and wet N2).

While some previous studies have noted that moisture may have an effect on ferroelectric behavior, they have not explored it in detai. In our case, we observed that the P-E loops were already stable under ambient conditions, indicating good ferroelectric performance even without specialized environments. We further investigated the effect of different gas atmospheres and found that the surrounding environment does influence the ferroelectric response, as shown in the figure below. Also, removing and reapplying the top electrode did not significantly alter the ferroelectric properties.

A screenshot of a computer screen

AI-generated content may be incorrect. 

A collage of diagrams

AI-generated content may be incorrect.

A group of graphs showing different types of cycles

AI-generated content may be incorrect.

A graph of a graph of a graph of a graph of a graph of a graph of a graph of a graph of a graph of a graph of a graph of a graph of a graph of

AI-generated content may be incorrect.

Comparison of polarization-electric field (P-E) hysteresis loops for pristine and field-cycled HZO samples measured under different environments: (a) Ambient, O₂, N₂, and CO₂ atmospheres, (b) Dry N₂ and Wet N₂ conditions; Corresponding PUND measurements are shown in (c) Ambient, O₂, N₂, and CO₂, and (d) Dry N₂ and Wet N₂ environments.

This suggests that while the electrode offers some protection to the ferroelectric layer, understanding the intrinsic surface behavior, especially in the absence of a top electrode is fundamentally important for gaining deeper insights into stability and device reliability.

- XPS Measurements on Samples Treated with Different Gases

Based on the reviewer’s valuable suggestion, we performed XPS analysis on HZO films treated under different atmospheric conditions to examine their surface chemistry and oxidation behavior. While we acknowledge that ambient pressure XPS (AP-XPS) would have provided more precise insights into real-time surface interactions, such facilities were not available to us at this stage. Therefore, we adopted an alternative approach by exposing the films to N2, O2, and CO2 atmospheres for several hours prior to XPS characterization. The samples were placed in desiccator jars that were evacuated and subsequently backfilled with the respective gases. They were maintained in these controlled environments for six hours. After exposure, the samples were briefly exposed to ambient air (approximately 5 minutes) before being mounted onto sample platens and loaded into the XPS system. Although we recognize that the reversion from a less ferroelectric to a more ferroelectric state upon atmospheric exposure is often rapid, the XPS still revealed distinct differences in surface chemistry under each condition, as shown below.

Figure (a) display the XPS spectra of Hf 4f, Zr 3d, and O 1s peaks recorded from the surfaces of HZO thin films. The Hf 4f and Zr 3d peaks correspond to the Hf⁴⁺ and Zr⁴⁺ oxidation states. 

Furthermore, the deconvolution of the O 1s XPS spectra reveals three distinct peaks, denoted as O1, O2, and O3. The O1 peak corresponds to lattice oxygen present in HfO2 and ZrO2. The O2 peak is attributed to weakly absorbed oxygen species, or non-lattice oxygen on the surface. The third peak, O3, is ascribed to hydroxyl groups adsorbed on the film surface. The O2 and O3 peaks reflect deviations from the ideal M:O stoichiometry, suggesting the formation of oxygen vacancies, related defects or adsorbed species.

The quantitative O:Hf and O:Zr ratios are summarized in Supplementary Figure 25(b). These results indicate that oxidation states and oxygen bonding vary with the atmospheric gas. In O2, both O:Hf and O:Zr ratios reach their highest values, and the binding energies for Hf 4f7/2 (~17.45 eV) and Zr 3d5/2 (~182.95 eV) are elevated, indicating enhanced oxidation. In N2, the oxidation is weakest, with the lowest atomic ratios and binding energies, suggesting more oxygen vacancies and sub-stoichiometric conditions, whereas CO2 gives intermediate level oxidation and binding energy. These findings reveal the role of processing atmosphere in determining the oxidation states of Hf and Zr, which in turn can influence the film's ferroelectric behavior and defect structure.

In the proposed model, we hypothesize that O2 effectively passivates oxygen vacancies, which leads to a faster back-transition process. The XPS data support this claim by showing significantly higher oxidation levels under O2 as pictorially represented in figure (c) Furthermore, when comparing N2 and CO2, both linear molecules, the model suggests that the higher stability in CO2 may stem from its ability to adopt a bent configuration under surface interactions, thereby providing more effective screening, as supported by previous literature. Interestingly, this flexibility is indirectly confirmed by XPS observations, which show that CO2 leads to higher oxidation than N2. The higher oxidation states of metal ions observed in the CO2-treated samples compared to those treated in N2 may be attributed to the bending of CO₂ molecules at oxygen vacancy sites as represented in figure c. This suggests that CO₂ can undergo bending upon interacting with charged surfaces, such as those exhibiting surface polarization, thereby contributing to charge screening, hence higher stability. 

A screenshot of a graph

AI-generated content may be incorrect.

A screenshot of a graph

AI-generated content may be incorrect.

A screenshot of a computer screen

AI-generated content may be incorrect.

Therefore, the XPS results support the proposed model. However, we believe that ambient pressure XPS (AP-XPS) will offer deeper insight into the dynamic surface processes involved, and this will be explored in future studies.

- Structural and PFM measurements on new Sample Annealed without Top Electrode:

We also performed structural and PFM measurements on a sample that was annealed without top tungsten layer to cross-check the impact of electrode etching. The behavior of this sample observed was similar to that of samples with etched top electrodes. Figure (b) shows the VAC sweeps of the sample measured under different atmospheric conditions (at RH of 49%, 18% and 6% in N2 atmosphere. The amplitude response was higher in controlled environments compared to ambient conditions. Figure (c) presents the PFM box-in-box switching in both ambient and controlled environments. As seen, domain switching was more distinct and stable in controlled environments highlighting that even in the film annealed with no top electrode and higher monoclinic phase, the observed behavior is similar.

A screenshot of a computer screen

AI-generated content may be incorrect.

(a) Comparison of XRD patterns of HZO samples annealed with and without a top W layer, (b) Variation in PFM amplitude response as a function of relative humidity for the sample annealed without the top W layer, and (c) Comparison of PFM phase and amplitude images of the sample annealed without the top W layer, measured under ambient and N₂ environments (RH = 7%).

Since the uncapped samples exhibited behavior comparable to those with etched electrodes, these results suggest that electrode etching may not have a significant impact on the surface functionalities.

Now, assuming that the composition is uniform, we strongly believe that the effect of the surrounding gas and humidity will still be noticeable. This is because the presence of oxygen vacancies plays a crucial role in stabilizing the orthorhombic phase and in determining the remnant polarization. Our findings show that a controlled environment helps in preserving oxygen vacancies from being passivated by atmospheric species. Moreover, different gases adsorb differently depending on the ferroelectric state of the film. Therefore, the behavior of the film is similar under such environmental conditions.

- Statistical Validation of Preferred Downward Polarization Orientation 

We crosschecked data from 20 different samples, and consistently observed this downward polarization behavior. Figure (b) presents PFM phase images from five different samples, all of which demonstrate the same preferred orientation.

(a) Box-in-box PFM phase and amplitude images of the HZO sample; (i) a negative voltage was applied over a 10 μm × 10 μm area, followed by a positive voltage on a 5 μm × 5 μm inner box; (ii) a positive voltage was applied over the 10 μm region, followed by a negative voltage on the 5 μm region. In both cases, the observed phase contrast indicates a preferred downward polarization orientation. (b) PFM phase images from five representative samples, all consistently showing the same downward polarization preference, confirming the reproducibility of this behavior across 20 films studied.
